# Mitochondrial Features and Expressions of MFN2 and DRP1 during Spermiogenesis in *Phascolosoma esculenta*

**DOI:** 10.3390/ijms232415517

**Published:** 2022-12-08

**Authors:** Xinming Gao, Binbin Feng, Daojun Tang, Chen Du, Congcong Hou, Shan Jin, Junquan Zhu

**Affiliations:** Key Laboratory of Applied Marine Biotechnology by the Ministry of Education and Key Laboratory of Marine Biotechnology of Zhejiang Province, School of Marine Sciences, Ningbo University, Ningbo 315211, China

**Keywords:** morphology, spermatid, invertebrate, mitofusin, dynamin-like protein

## Abstract

Mitochondria can fuse or divide, a phenomenon known as mitochondrial dynamics, and their distribution within a cell changes according to the physiological status of the cell. However, the functions of mitochondrial dynamics during spermatogenesis in animals other than mammals and fruit flies are poorly understood. In this study, we analyzed mitochondrial distribution and morphology during spermiogenesis in Sipuncula (*Phascolosoma esculenta*) and investigated the expression dynamics of mitochondrial fusion-related protein MFN2 and fission-related protein DRP1 during spermiogenesis. The mitochondria, which were elliptic with abundant lamellar cristae, were mainly localized near the nucleus and distributed unilaterally in cells during most stages of spermiogenesis. Their major axis length, average diameter, cross-sectional area, and volume are significantly changed during spermiogenesis. *mfn2* and *drp1* mRNA and proteins were most highly expressed in coelomic fluid, a spermatid development site for male *P. esculenta*, and highly expressed in the breeding stage compared to in the non-breeding stage. MFN2 and DRP1 expression levels were higher in components with many spermatids than in spermatid-free components. Immunofluorescence revealed that MFN2 and DRP1 were consistently expressed and that MFN2 co-localizes with mitochondria during spermiogenesis. The results provide evidence for an important role of mitochondrial dynamics during spermiogenesis from morphology and molecular biology in *P. esculenta*, broadening insights into the role of mitochondrial dynamics in animal spermiogenesis.

## 1. Introduction

Mitochondria are an important type of organelle involved in adenosine triphosphate (ATP) synthesis, regulation of cell metabolism, signal transduction, and apoptosis [1,2]. The distribution of mitochondria in a cell is dynamic and abundant in areas where cell energy requirements are concentrated [3]. As in neural cells, mitochondrial distribution is variable with the influence of neuronal activity and synaptic location [4]. In addition, the morphology of mitochondria is also closely related to the physiological state of the cell, which changes according to cell life activities. For example, under excess nutrition, mitochondria undergo fragmentation and miniaturization, and under poor nutrition, mitochondria tubularize and grow larger [5].

Mitochondrial fusion and fission, known as mitochondrial dynamics, have a large impact on mitochondrial morphology, distribution, and physiology [6,7]. Enhanced fusion or reduced fission is generally accompanied by tubularization and enlargement of mitochondria, which are not conducive to the rational distribution of mitochondria within the cell; in contrast, reduced fusion or enhanced fission leads to mitochondrial fragmentation and miniaturization, favoring transport and rational distribution of mitochondria within the cell [7]. Mitofusin-2 (MFN2) and dynamin-related protein 1 (DRP1), two dynamin family members, have been reported to play important roles in mitochondrial fusion and fission, respectively. For example, knockdown of *mfn2* results in mitochondrial non-fusion and fragmentation [8,9], while knockdown of *drp1* increases the mitochondria volume and diminishes mitochondrial oxidative phosphorylation capacity and oxidative damage [10].

Spermatogenesis is closely related to the fertility of males and is an important part of reproductive biology research. During spermatogenesis, spermatogonia first differentiate into primary spermatocytes, which develop into secondary spermatocytes after the first meiosis cycle and into spermatids after the second meiosis cycle. During this complex and subtle cytological process (spermiogenesis), the spermatids eventually develop into mature sperm [11].

Mitochondria play important roles in spermatogenesis [12]. For example, abnormal mitochondrial membrane potential and mitochondrial DNA mutations affect spermatogenesis, sperm quality, and animal fertility [13,14,15,16]. Furthermore, mitochondrial distribution and morphology are changed in spermatogenic cells to promote spermatogenesis [12,17,18]. These morphological changes implicate a role for mitochondria dynamics in spermatogenesis. Indeed, in mice (*Mus musculus*), knockdown of *mfn1* and/or *mfn2* leads to abnormal spermatogenesis and male infertility [19]; in fruit flies, mutation of *drp1* causes an abnormal accumulation of mitochondria in primary spermatocytes [20]. Furthermore, mitochondria are not normally assigned to the daughter cells during meiosis, resulting in some spermatids without mitochondria [20]. However, mitochondrial dynamics and its functions and regulatory mechanisms in spermatogenesis in other animals have not been reported.

*Phascolosoma esculenta* belongs to the phylum Sipuncula and family Phascolosomatidae [21]. It is an economically important species for artificial culture in the southeast coast of China. However, the reproductive biology research and artificial breeding technology of this species are insufficient, which means that the larvae of *P. esculenta* used in aquaculture come entirely from the wild. This situation hinders the continued development of the *P. esculenta* aquaculture industry. Studies of the mechanism of gametogenesis, therefore, are requisite for improving and perfecting the artificial breeding technology for providing a continuous supply of larvae for aquaculture. Currently, the researchers have found that its spermatogenesis is completed in the testis (in the basal part of the retractor muscle) and coelomic fluid [22]. Mitosis (spermatogonia) and meiosis (spermatocytes) mainly occur in the testis, while spermiogenesis is mainly completed in the coelomic fluid [22]. However, the effects of mitochondria and their physiological functions on spermatogenesis have not been reported.

In this study, we analyzed mitochondrial distribution and morphology and investigated the expression dynamics of the mitochondrial fusion-related protein MFN2 and fission-related protein DRP1 during spermiogenesis. The results indicate that the mitochondrial dynamics play roles during spermiogenesis in *P. esculenta* and provide a theoretical reference for promoting male germ cell maturation.

## 2. Results

### 2.1. Mitochondrial Ultrastructure during Spermiogenesis

**Figure 1:**
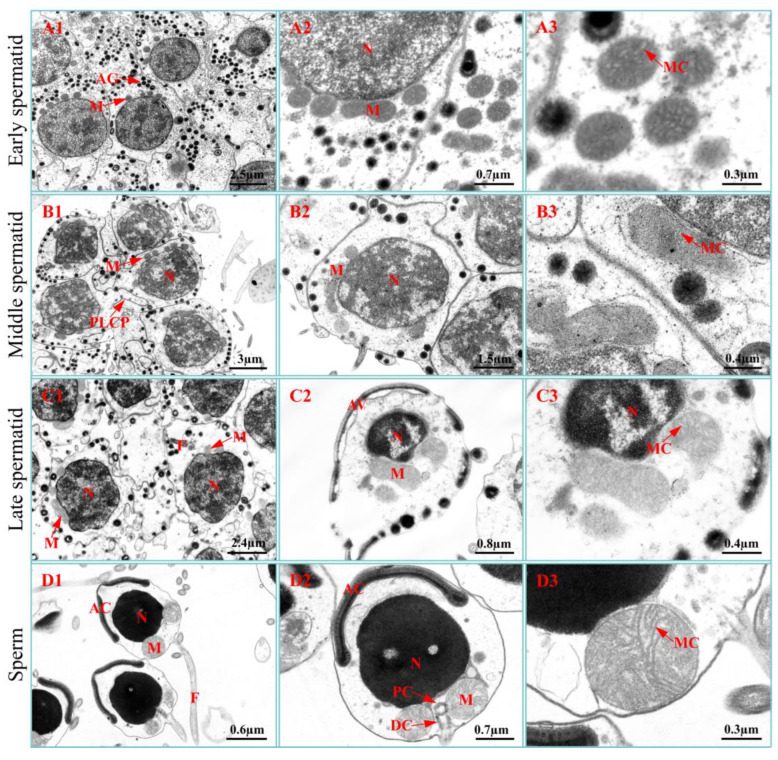
Ultrastructure of spermiogenesis. (**A1**–**A3**) Early spermatid, (**B1**–**B3**) middle spermatid, (**C1**–**C3**) late spermatid, and (**D1**–**D3**) sperm. AC: acrosome, AG: acrosomal granule, DC: distal centriole, F: flagellum, M: mitochondrion, MC: mitochondrial cristae, N: nucleus, PC: proximal centriole, PLCP: pseudopodia-like cytoplasmic protrusion.

In early spermatids, the mitochondria were randomly distributed in the perinuclear cytoplasm or primarily on one side of the spermatid (Figure 1 and Figure S1). The mitochondria were predominantly oval-shaped, with abundant lamellar cristae (Figure 1). Their major axis length, minor axis length, average diameter, eccentricity, cross-sectional area, and volume were 0.49 ± 0.11 µm, 0.39 ± 0.07 µm, 0.44 ± 0.08 µm, 0.56 ± 0.16, 0.18 ± 0.06 µm^2^, and 0.04 ± 0.02 µm^3^, respectively (Table S1). 

In middle spermatids, the mitochondria had a distribution similar to that in early spermatids (Figure 1 and Figure S1). The mitochondria were also primarily oval-shaped, with abundant lamellar cristae (Figure 1). Their major axis length, minor axis length, average diameter, eccentricity, cross-sectional area, and volume were 0.69 ± 0.29 µm, 0.43 ± 0.09 µm, 0.56 ± 0.16 µm, 0.70 ± 0.14, 0.28 ± 0.15 µm^2^, and 0.07 ± 0.05 µm^3^, respectively (Table S1). 

In late spermatids, the mitochondria were distributed unilaterally (Figure 1 and Figure S1). The mitochondria were primarily elliptical, with abundant lamellar cristae (Figure 1). Their major axis length, minor axis length, average diameter, eccentricity, cross-sectional area, and volume were 0.59 ± 0.28 µm, 0.42 ± 0.06 µm, 0.51 ± 0.15 µm, 0.57 ± 0.18, 0.23 ± 0.13 µm^2^, and 0.06 ± 0.04 µm^3^, respectively (Table S1). 

In sperm, the mitochondria and centrosomes constitute the sperm midpiece (Figure 1, Figures S1 and S2). The mitochondria were approximately circular with abundant lamellar cristae (Figure 1). Their major axis length, minor axis length, average diameter, eccentricity, cross-sectional area, and volume were 0.70 ± 0.10 µm, 0.62 ± 0.08 µm, 0.66 ± 0.09 µm, 0.43 ± 0.12, 0.36 ± 0.09 µm^2^, and 0.08 ± 0.03 µm^3^, respectively (Table S1).

**Figure 2:**
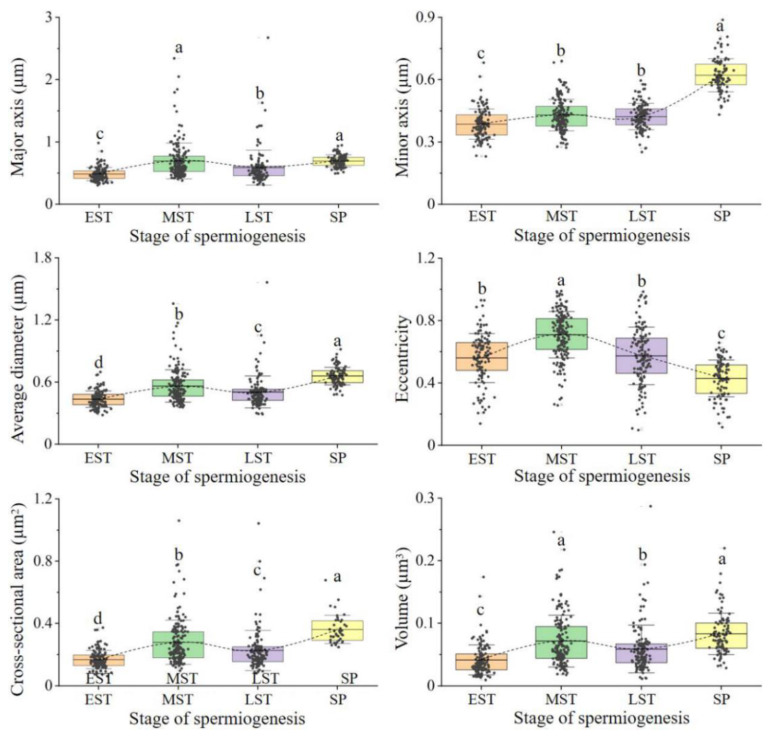
Mitochondrial major axis length, minor axis length, average diameter, eccentricity, cross-sectional area, and volume during spermiogenesis. Different letters above columns indicate significant differences (*p* < 0.05). EST: early spermatid, MST: middle spermatid, LST: late spermatid, SP: sperm.

As shown in Figure 2 and Figure S3 and Table S1, the mitochondrial major axis length, average diameter, cross-sectional area, and volume first increased, then decreased, and then increased during spermiogenesis, the minor axis length continuously increased, and the eccentricity tended to increase and then decrease. The volumes of mitochondria in the middle spermatids, late spermatids, and sperm were about 1.75, 1.5, and 2 times more, respectively, than those in the early spermatids. The mitochondria became elongated and then round. 

### 2.2. Expression Dynamics of MFN2 and DRP1 during Spermiogenesis

#### 2.2.1. Full-Length cDNA of *Pe*-*mfn2* and *Pe*-*drp1* and Characteristics of Their Protein Structures

As shown in Figures S4 and S5, the full lengths of the cDNA of *P. esculenta mfn2* and *drp1* (*Pe-mfn2* and *Pe-drp1*, respectively) were 3362 and 3643 bp, respectively, and consisted of 206 and 91 bp 5′ untranslated regions, 879 and 1398 bp 3′ untranslated regions, and 2277 and 2154 bp open-reading frames encoding 759 and 718 amino acids (GenBank accession numbers of *Pe-mfn2* and *Pe-drp1* are OP067227 and OP067228, respectively). The predicted molecular weights of *Pe*-MFN2 and DRP1 were 86.33 and 80.5 kDa, respectively, and their theoretical isoelectric points were pH 6.52 and 6.66, respectively. The contents of Ala, Arg, Glu, Leu, Lys, Ser, and Val in MFN2 were higher, accounting for 7.4%, 6.5%, 9.0%, 9.0%, 7.4%, 7.4%, and 6.9%, respectively, of the total amino acids. The contents of Ala, Arg, Glu, Ile, Leu, Ser, Thr, and Val were higher in DRP1, accounting for 6.0%, 6.7%, 7.7%, 7.4%, 10.3%, 7.0%, 6.1%, and 7.1%, respectively, of the total amino acids. *Pe*-MFN2 includes a GTPase domain, two helical bundle regions (HB1 and HB2), and two transmembrane domains (Figure S6A,B). *Pe*-DRP1 includes a GTPase domain, a bundle signaling element, a stalk, and a variable domain (also known as B-insert; Figure S6C,D). Fourteen and twenty GTP binding sites were identified in the GTPase domain in MFN2 and DRP1, respectively.

As shown in Figure S7, the consensus positions and identity positions of *Pe*-MFN2 and MFN2 in *M. musculus*, *Python bivittatus*, *Xenopus tropicalis*, *Danio rerio*, *Strongylocentrotus purpuratus*, *Crassostrea gigas*, and *Daphnia magna* were, respectively, 65.7% and 49.9%, 64.4% and 49.2%, 66.1% and 50.8%, 65.6% and 51.59%, 63.7% and 48.49%, 65.4% and 53.19%, and 65.4% and 50.29%. The consensus positions and identity positions of *Pe*-DRP1 and DRP1 in *Homo sapiens*, *Gallus gallus*, *Xenopus laevis*, *D. rerio*, *Drosophila melanogaster*, *C. gigas*, and *Caenorhabditis elegans* were, respectively, 78.5% and 67.6%, 78.1% and 67.4%, 77.7% and 67.3%, 78.9% and 68.0%, 78.1% and 66.5%, 80.8% and 71.9%, and 72.9% and 61.2% (Figure S8). Furthermore, as shown in Figures S9 and S10, high similarity and consistency were identified between MFN2 and DRP1 from different species, including vertebrates and vertebrates, invertebrates and invertebrates, and vertebrates and invertebrates, indicating that the MFN2 and DRP1 proteins are highly homologous between species and were conserved during animal evolution. *Pe*-MFN2 was determined to be most closely related to mollusk MFN2, and *Pe*-DRP1 was found to be most closely related to Annelida DRP1 (Figures S11 and S12). 

#### 2.2.2. High Expression of MFN2 and DRP1 in Coelomic Fluid

**Figure 3:**
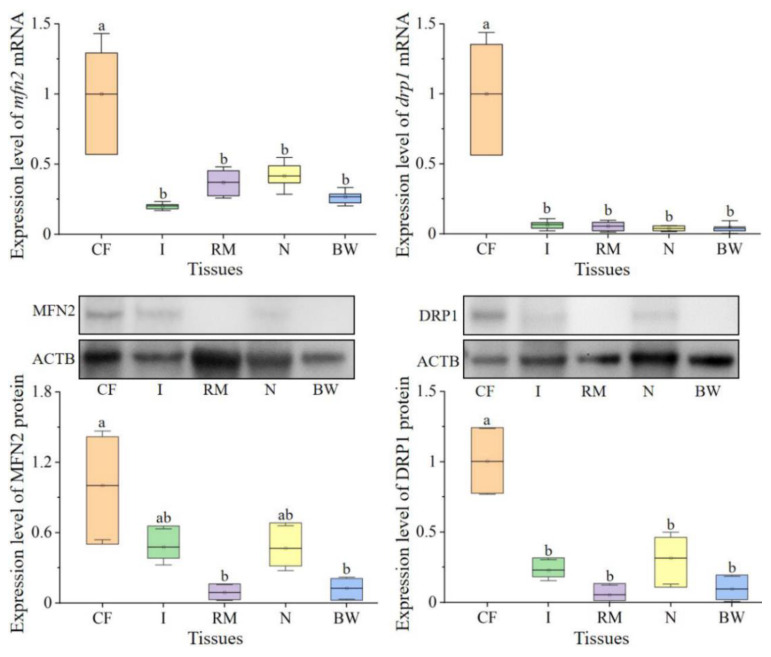
Expression of *mfn2* and *drp1* mRNA and proteins in tissues. The different letters above columns indicate significant differences (*p* < 0.05). BW: body wall, CF: coelomic fluid, I: intestine, N: nephridium, RM: retractor muscle.

To detect the expression of *mfn2* and *drp1* mRNA in different tissues, the qPCR experiments were performed. As shown in Figure 3, *mfn2* and *drp1* mRNA were expressed in the coelomic fluid, intestine, retractor muscle, body wall, and nephridium, with the highest expression in coelomic fluid. 

To detect the expression of MFN2 and DRP1 proteins, we produced MFN2 and DRP1 antibodies. As shown in Figure S13, we obtained recombinant MFN2 and DRP1. After rats were immunized with the purified recombinant proteins, specific rat anti-*Pe*-MFN2/DRP1 antibodies were obtained (Figure S13). Using WB, we found that MFN2 and DRP1 were expressed at high levels in coelomic fluid (Figure 3).

#### 2.2.3. High Expression of MFN2 and DRP1 during Breeding Season

**Figure 4:**
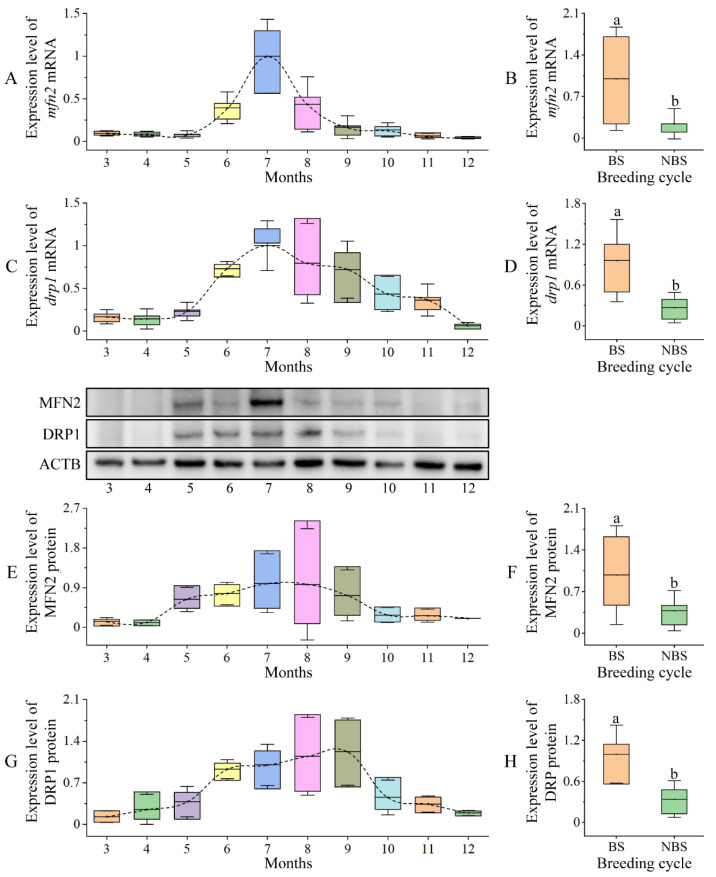
Expression of *mfn2* and *drp1* mRNA and protein in the coelomic fluid of male *P. esculenta* during different months and breeding cycle stages. (**A**,**C**,**E**,**G**) Expression of *mfn2* and *drp1* mRNA and protein in coelomic fluid during different months. Expression first increased and then decreased. (**B**,**D**,**F**,**H**) Expression of *mfn2* and *drp1* mRNA and protein in coelomic fluid during different breeding cycle stages. The different letters above columns indicate significant differences (*p* < 0.05). BS: breeding stage, NBS: non-breeding stage.

The coelomic fluid is the site of spermatid development. After detecting the high expression of *mfn2* and *drp1* genes in this tissue, we curiously analyzed the expression of *mfn2* and *drp1* mRNA and proteins in the coelomic fluid during the reproductive cycle. As shown in Figure 4, *mfn2* and *drp1* mRNA and proteins were detected in the coelomic fluid of male *P. esculenta* from March to December, and the expression levels first increased and then decreased. We analyzed samples from the breeding stage (July to September) and the non-breeding stage (March to June and October to December) and found that the expression levels of *mfn2* and *drp1* mRNA and proteins in the breeding stage were significantly higher than those in the non-breeding stage (Figure 4).

#### 2.2.4. High Expression of MFN2 and DRP1 in Coelomic Fluid Spermatid

After detecting the high expression of *mfn2* and *drp1* genes in coelomic fluid in the breeding season, we separated the components in coelomic fluid to analyze in which component the MFN2 and DRP1 proteins were mainly distributed. Cells in the breeding stage in coelomic fluid were divided into four layers through centrifugation (Figure S14). The first and second layers were removed separately and named spermatid component 1 (ST1) and spermatid component 2 (ST2), respectively. Since layer 4 had few cells, layers 3 and 4 were sampled as a single layer and named as the spermatid-free component (STF).

**Figure 5:**
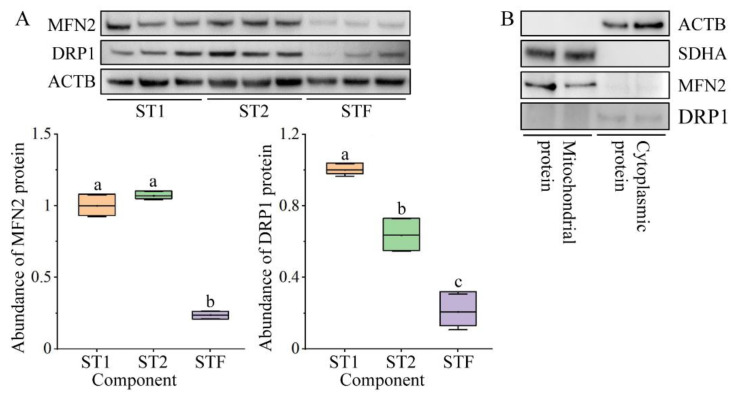
Expression of MFN2 and DRP1 in different components of coelomic fluid and their subcellular localization in early spermatid. (**A**) Expression of MFN2 and DRP1 in different components of coelomic fluid. The different letters above columns indicate significant differences (*p* < 0.05). ST1 and ST2: components consisting of many spermatids; STF: spermatid-free components. (**B**) Distribution of MFN2 and DRP1 in spermatid mitochondria and cytoplasm from which mitochondria were removed.

The MFN2 and DRP1 were more highly expressed in components with many spermatids and had lower expression in spermatid-free components (Figure 5A).

#### 2.2.5. Localization of MFN2 and DRP1 in Early Spermatids

To examine the subcellular localization of MFN2 and DRP1 in spermatids, we isolated the mitochondria and cytoplasm. As shown in Figure 5B, MFN2 was detected in the mitochondrial total protein, while DRP1 was mainly detected in the cytoplasm. These results suggest that MFN2 is mainly localized to mitochondria and DRP1 is mainly found in the cytoplasm of early spermatids.

#### 2.2.6. Consistent Expression of MFN2 and DRP1 during Spermiogenesis

**Figure 6:**
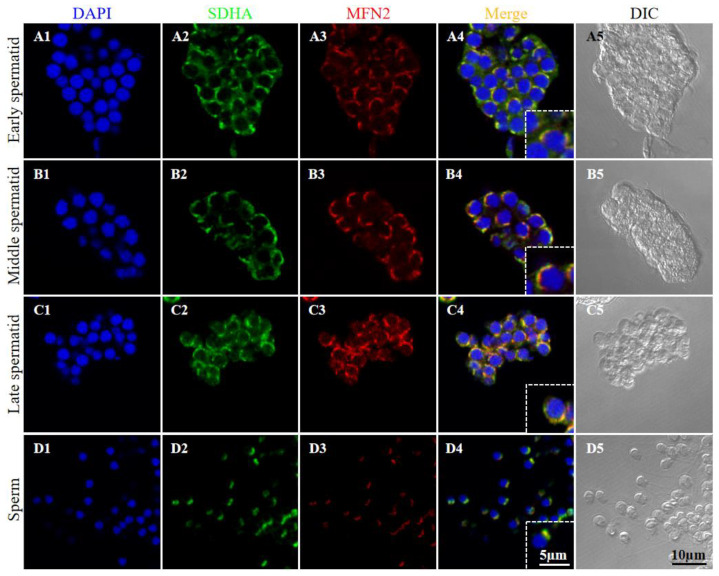
Expression of *Pe*-MFN2 and its co-localization with succinate dehydrogenase (SDHA) during spermiogenesis in *P. esculenta*. (**A1**–**A5**) Early spermatid, (**B1**–**B5**) middle spermatid, (**C1**–**C5**) late spermatid, and (**D1**–**D5**) sperm. Blue indicates nuclei stained with DAPI, green indicates SDHA, a mitochondrial marker, and red indicates MFN2. DIC, differential interference contrast imaging.

To analyze whether the MFN2 and DRP1 may play roles in spermiogenesis, we detected the expression dynamics of MFN2 and DRP1 during spermiogenesis using the immunofluorescence (IF) experiment. As shown in Figure 6, MFN2 was consistently detected and colocalized with SDHA in mitochondria during spermiogenesis. In early and middle spermatids, MFN2 and mitochondria were distributed in the cytoplasm randomly or unilaterally in cells (Figure 6(A1–A5,B1–B5)). In late spermatids, MFN2 and mitochondria were clustered on one side of the cells (Figure 6(C1–C5)). In sperm, MFN2 and mitochondria were located in the midpiece (Figure 6(D1–D5)).

**Figure 7:**
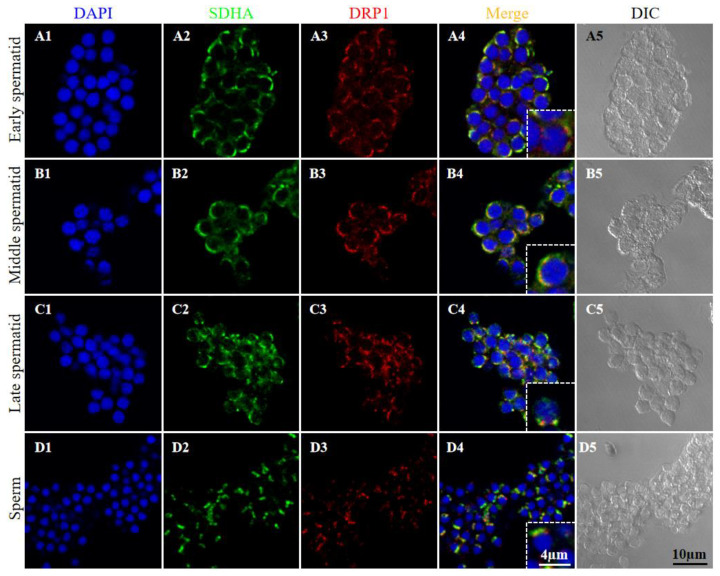
Expression of DRP1 during spermiogenesis in *P. esculenta*. (**A1**–**A5**) Early spermatid, (**B1**–**B5**) middle spermatid, (**C1**–**C5**) late spermatid, and (**D1**–**D5**) sperm. Blue indicates nuclei stained with DAPI, green indicates SDHA, a mitochondrial marker, and red indicates DRP1. DIC, differential interference contrast imaging.

As shown in Figure 7, the expression of DRP1 was detected during spermiogenesis. In early, middle, and late spermatids, DRP1 was distributed in the cytoplasm near the mitochondria (Figure 7(A1–A5,B1–B5,C1–C5)). In sperm, DRP1 localized to the midpiece (Figure 7(D1–D5)).

## 3. Discussion

### 3.1. Mitochondrial Distribution during Spermiogenesis

The distribution of mitochondria during spermatogenesis in several animals, including (but not limited to) Mollusca [23], Arthropoda [24], Pisces [25], Reptile [26], and Mammalia [27,28], has been reported. In *Octopus tankahkeei*, the mitochondria are randomly distributed in the cytoplasm of early spermatids: some are polarized to the tail to form the midpiece, and some migrate to the top of the (eventual) sperm head and are discarded with the cytoplasm in the late stage of spermiogenesis [23]. In *Opsariichthys bidens*, the mitochondria are gradually distributed in the cytoplasm on the side of tail formation during spermiogenesis until six to eight mitochondria are clustered in the midpiece [25]. In *Pelodiscus sinensis*, mitochondria are randomly distributed in early spermatids and are gradually distributed near the membrane during spermatid development. The mitochondria are distributed along the outer dense fibers in the midpiece in the late maturation phase [26]. In *Rattus norvegicus*, mitochondria are distributed in the cell periphery in the Golgi phase, cap phase, and early acrosome phases of spermiogenesis and move to the flagellum in the late acrosome and early maturation phases. Finally, mitochondria are distributed along the outer dense fibers in the midpiece in the late maturation phase [27]. In *P. esculenta*, most mitochondria are distributed close to the nucleus and on the side of tail formation; in sperm, about six mitochondria, along with centrosomes, constitute the midpiece.

The distribution of mitochondria is closely related to the energy demand of the cell, and areas with a high energy demand tend to gather more mitochondria [3]. The distribution characteristics of mitochondria during spermatogenesis may also reflect the variability in energy requirements in different parts of spermatogenic cells, reflecting the cellular processes in which mitochondria are involved. In *P. esculenta*, the perinuclear distribution of mitochondria suggests that mitochondria may play an important role in chromatin concentration and sperm nuclear morphogenesis. In addition, the distribution of mitochondria on the side of tail formation may be related to mitochondria as a component of the sperm midpiece. Alternatively, tail formation requires a lot of energy, and the distribution of mitochondria on this side may facilitate the energy supply for the material transport required for tail formation and tail morphology construction.

### 3.2. Morphological Evidence for the Role of Mitochondrial Dynamics during Spermatogenesis

The morphological features of mitochondria during spermatogenesis in several animals, including Arthropoda [24], Pisces [29], Reptile [26], and Mammalia [17], have also been reported. In *D. melanogaster*, small mitochondria aggregate and eventually fuse to form two large mitochondria (which constitute a nebenkern in spermatid) during spermatogenesis [24]. In *Pampus argenteus*, the mitochondria are diverse in size and morphology (including round, oval, elliptical, short, or irregular), and while cristae are abundant, their distribution in the mitochondria is irregular in spermatogonia and spermatocytes. In spermatids, the mitochondria are generally uniform in size and usually elliptical, and the cristae are regular and evenly distributed. In sperm, the mitochondria are approximately circular and uniform in size [29]. In *Pelodiscus sinensis*, the mitochondria are round in the early spermatid phase, gradually elongate in the middle spermatid phase, fuse in the late spermatid phase, and eventually become onion-shaped in sperm [26]. In *R. norvegicus*, orthodox-type mitochondria are usually found in spermatogonia and preleptotene and leptotene spermatocytes, intermediate-type mitochondria are usually found in synaptene spermatocytes, late spermatids, and sperm, and condensed-type mitochondria are usually found in pachytene spermatocytes, secondary spermatocytes, and early spermatids [12,17]. In addition, the mitochondria are usually spherical, with small volumes in spermatogonia and primary spermatocytes in prophase. Mitochondria elongate in primary spermatocytes at the pachytene stage and are fragmented in spermatids [30]. In *P. esculenta*, the mitochondria are oval-shaped with small volumes in early spermatids, and the major axis, minor axis, and volume significantly increase in the middle spermatid phase. In late spermatid, the mitochondria have a significantly reduced volume and a more spherical shape compared with those in the middle spermatid phase. In sperm, the mitochondria are nearly round and have approximately twice the volume of mitochondria in early spermatids. These results indicate that mitochondrial fusion and fission occur during spermiogenesis, which affect mitochondrial morphology and volume during spermiogenesis.

Mitochondrial morphology may change in response to the physiological needs of spermatogenic cells. Spermatogonia have strong glycolytic capacity, while spermatocytes and spermatids synthesize ATP mainly by oxidative phosphorylation. The changes, therefore, may improve oxidative phosphorylation and promote spermatogenic cell development [12,31,32,33]. In *P. esculenta*, the changes in mitochondrial morphology and volume may be related to spermiogenesis and conducive to spermatid differentiation. Mitochondrial fusion usually favors ATP synthesis [5]. The mitochondria fusion into large mitochondria in sperm may be more conducive to ATP synthesis. Measurement of the oxidative phosphorylation level in spermatids at each level and determining its relationship with mitochondrial morphology may reveal the physiological significance of mitochondrial dynamics in *P. esculenta*.

### 3.3. Molecular Biology Evidence for a Role of Mitochondrial Dynamics during Spermatogenesis

MFNs and DRP1 are the most important proteins for mitochondrial fusion and fission, respectively. Insufficient MFNs will cause mitochondrial fragmentation, while insufficient DRP1 leads to mitochondrial tubularization and enlargement. For instance, in mouse embryo fibroblasts, the absence of either MFN1 or MFN2 results in mitochondrial fragmentation, with reduced membrane potential and a weakened oxidative phosphorylation ability, which affects cell respiration and growth [34]. In *M. musculus*, adipose tissue-specific knockout of *mfn2* shortens the mitochondrial length, reduces the cristae number, and weakens the activity of respiratory chain complexes I and III [35]. Liver tissue-specific knockdown of *mfn2* results in mitochondrial fragmentation, weakened respiratory chain complex activity, and increased H_2_O_2_ content in liver cells [36]. In *C. elegans*, *drp1* mutation leads to mitochondrial tubularization, while overexpression results in mitochondrial fragmentation. Subcellular localization analyses have found that DRP1 is localized at mitochondrial fission sites [37]. In *M. musculus*, the absence of DRP1 results in a reduced mitochondria number and an increased mitochondria volume in live cells [38]. In this study, we found that *Pe*-MFN2 and *Pe*-DRP1 have conserved structural features, including highly similar primary structure, protein domain, and GTP binding sites, and that, like MFN2 and DRP1, they mainly localize in mitochondria and cytolymph, respectively. These features suggest that *Pe*-MFN2 and *Pe*-DRP1 have functions in mitochondrial fusion and fission that are similar to those of their homologous proteins in mammals.

Mitochondrial fusion and fission have been reported to play important roles in animal spermatogenesis. For instance, low expression of *fzo*, which in *Drosophila* codes for a protein homologous to MFNs, causes failure of spermatid mitochondrial fusion, resulting in male sterility [24]. MFN2 is highly expressed in the spermatid of *R. norvegicus* [39], and knockdown of *mfn1*, *mfn2*, or *mfns* results in abnormal spermatogonia differentiation and spermatocyte development and causes male sterility [19,40]. Knockdown of the gene encoding the mitochondrial fusion-related protein MitoPLD causes meiotic arrest and male sterility in *M. musculus* [41,42]. The functions of mitochondrial fission in spermatogenesis in animals have also been previously reported. In *Drosophila*, insufficient DRP1 causes the aberrant aggregation of mitochondria in primary spermatocytes, and mitochondria are not assigned to the daughter cells during meiosis [41,42], indicating that mitochondrial fission is involved in the correct distribution of mitochondria in spermatogenic cells. In addition, insufficient DRP1 also increases cellular reactive oxygen species content and activates epithelial growth factor receptor signaling, prompting premature differentiation of germ stem cells and spermatogonia in *Drosophila* [43]. In *R. norvegicus*, DRP1 is more highly expressed in the testis in adolescent and mature individuals and more highly expressed in round and elongated spermatids [39]. In addition, insufficient mitochondrial fission factor causes excess mitochondrial fusion in round spermatids, abnormal organization of mitochondria in the midpiece in elongating spermatids, and discontinuous distribution of mitochondria in sperm mitochondrial sheaths, and leads to sperm with reduced motility, fertility, and respiratory chain complex IV activity [44]. Insufficient mitochondrial fission 1 protein results in spermatid arrest with multinucleation, fragmented acrosomes, and infertility [45]. In this study, we found that MFN2 and DRP1 were highly expressed in the coelomic fluid in which spermiogenesis occurs. Furthermore, their expression levels were higher during the breeding stage than the non-breeding stage. WB analyses indicated that MFN2 and DRP1 were mainly found in spermatids in coelomic fluid, and IF experiments showed that MFN2 and DRP1 were expressed consistently during spermiogenesis. These results imply that mitochondrial fusion and fission play roles in spermiogenesis in *P. esculenta*. Artificial regulation of mitochondrial dynamics may be a potential idea to regulate the process of spermiogenesis, thus promoting artificial breeding technology to obtain larvae of *P. esculenta* for aquaculture.

## 4. Materials and Methods

### 4.1. Animals and Tissues

From 2018 to 2021, 100 *P. esculenta* individuals of 3.5–5.5 g were collected monthly from the Xizhou in Ningbo City, China. The coelomic fluid (mainly including the germ cell agglomerate, blood cells, and a few granular cells), body wall, intestine, retractor muscle, and nephridium were sampled from 12 males and immediately put into liquid nitrogen, then kept in a refrigerator at −80 °C until RNA and protein extraction. 

To obtain spermatids at different stages, we conducted artificial induction by running water to promote spermiogenesis of *P. esculenta* from July to September according to a previously described method [22]. Coelomic fluids were extracted before and after artificial induction and fixed in 2.5% glutaraldehyde fix solution (Solarbio, Beijing, China) and 4% paraformaldehyde fix solution (Beyotime, Shanghai, China) for transmission electron microscopy and immunofluorescence experiments, respectively. 

The coelomic fluid collected from July to September was separated into components by centrifugation using the following method: coelomic fluid samples were placed in 1.5 mL centrifuge tubes, centrifuged at 3000× *g* (4 °C) for 2 min, and washed twice with phosphate-buffered saline (PBS) (with centrifugation for 2 min after each wash). The cells in each cell layer were observed to identify components and then stored in a refrigerator at −80 °C until protein extraction. In addition, we separated the mitochondria and cytoplasm in which the mitochondria were removed from the spermatid components using a tissue mitochondria isolation kit (Beyotime).

### 4.2. Transmission Electron Microscope

The experiments were implemented as described in our previous report [23]. Briefly, fresh coelomic fluid samples were fixed in 2.5% glutaraldehyde fix solution (Solarbio) at 4 °C for about 24 h. The samples were then washed three times with 0.1 M PBS for 15 min each time and immediately fixed in 1% osmic acid in 0.1 M PBS in the dark at 4 °C for 1.5 h. The samples were dehydrated in 30% ethanol for 15 min, 50% ethanol for 15 min, 70% ethanol for 15 min, 90% ethanol for 15 min, 90% acetone for 15 min, and 100% acetone 3 times for 15 min each time. Finally, the samples were embedded in epoxy resin and cut into ultrathin sections. The sections were stained with uranyl acetate, counterstained with lead citrate, and then observed and photographed using an H-7650 transmission electron microscope (Hitachi, Tokyo, Japan) with a digital integrated camera and original software.

### 4.3. Immunofluorescence

The experiments were implemented as described in our previous report [23]. Briefly, fresh coelomic fluid samples were fixed in 4% paraformaldehyde fix solution (Beyotime) and embedded in an optimal cutting temperature compound (Sakura, Torrance, CA, USA) to obtain embedding blocks. The blocks were then sliced into 5 μm-thick sections and dried for 10 min at 20 °C. Subsequently, the sections were incubated in 0.3% Triton X-100 in PBS buffer (PBST) for 15 min, 5% bovine serum albumin in PBS buffer for 1.5 h, and primary antibody in 3% bovine serum albumin in PBS buffer for 12 h (4 °C); then, washed three times in 0.1% PBST buffer for 45 min, incubated in secondary antibody in 3% bovine serum albumin in PBS buffer for 1.5 h (25 °C), washed six times in 0.1% PBST for 90 min, and then stained with diamidino-phenyl-indole buffer (DAPI; Beyotime) for 5 min. Finally, the sections were mounted and immediately observed and photographed with a Zeiss laser scanning confocal microscope (LSM880, Carl Zeiss, Jena, Germany).

The primary antibodies used in this study were rabbit anti-succinate dehydrogenase (SDHA) antibody (Beyotime), rat anti-MFN2 antibody, and rat anti-DRP1 antibody. The immunogen for manufacturing rabbit anti-SDHA antibody was 385E-664Y. We aligned the immunogen amino acid sequence of the SDHA antibody to the homologous amino acid sequence in *P. esculenta* SDHA (Figure S15) and validated the specificity of this antibody by Western blotting (WB) (see Section 4.7) (Figure S16). The rat anti-MFN2 antibody and rat anti-DRP1 antibody were self-made antibodies obtained as described in our previous study [46]. The immunogens for manufacturing MFN2 and DRP1 antibodies were 223A-382V and 39S-225D, respectively, and were obtained by prokaryotic expression. The specificity of the antibodies was detected by WB. 

For the detection of mitochondrial distribution, the primary antibody was rabbit anti-SDHA antibody (Beyotime, 1:75 dilution), and the secondary antibody was Alexa Fluor 488-labeled goat anti-rabbit IgG (H+L; Beyotime; 1:500 dilution). For the detection of expression and co-localization of MFN2 and SDHA during spermiogenesis, the primary antibodies were rat anti-MFN2 antibody (1:75 dilution) and rabbit anti-SDHA antibody (Beyotime, 1:75 dilution), and the secondary antibodies were Cy3-labeled goat anti-rat IgG (H+L; Beyotime; 1:500 dilution) and Alexa Fluor 488-labeled goat anti-rabbit IgG (H+L; Beyotime; 1:500 dilution). For the detection of DRP1 expression during spermiogenesis, the primary antibody was rat anti-DRP1 antibody (1:75 dilution), and the secondary antibody was Cy3-labeled goat anti-rat IgG (H+L; Beyotime; 1:500 dilution).

### 4.4. Full-Length cDNA Cloning of mfn2 and drp1

#### 4.4.1. RNA Extraction and Reverse Transcription

TRIzon Reagent (Cwbio, Beijing, China) was used according to the manufacturer’s instructions for total RNA extraction from coelomic fluid. The PrimeScript^®^ RT Reagent Kit (Takara, Dalian, China) was used for reverse transcription to clone the intermediate segment sequences. The Smart RACE cDNA Amplification Kit (Takara) and 3′full RACE Amplification Kit (Takara) were used for reverse transcription for 5′ rapid amplification of cDNA ends (RACE) and 3′ RACE, respectively.

#### 4.4.2. Full-Length cDNA Cloning

The intermediate segment sequences of *mfn2* and *drp1* cDNA came from transcriptome sequencing of *P. esculenta*. Primer Premier 5.0 software was used to design primers (Tables S2 and S3). The intermediate segment sequences were verified by PCR using the following procedure: 1 cycle at 94 °C for 5 min, 35 cycles at 94 °C for 30 s, 55 °C for 30 s, and 72 °C for 1.5 min, and 1 cycle at 72 °C for 5 min. Touchdown PCR for 3′ and 5′ RACE was conducted as follows: 1 cycle at 94 °C for 5 min, 8 cycles at 94 °C for 30 s, 63 °C (3′ RACE)/69.5 °C (5′ RACE) for 30 s (decrease rate 0.5 °C/cycle), and 72 °C for 2 min, 27 cycles at 94 °C for 30 s, 59 °C (3′ RACE)/65.5 °C (5′ RACE) for 30 s, and 72 °C for 2 min, and then a final extension at 72 °C for 5 min. The PCR products were separated, identified, and sequenced, as previously described [47]. The full-length cDNA sequences of *mfn2* and *drp1* were obtained by stitching the intermediate segment sequences and the cDNA sequence from 3′/5′ RACE.

### 4.5. Analyses of Protein Structures and Properties

The primary structure, molecular weight, isoelectric point, transmembrane domains, tertiary structure, and GTP binding sites were predicted by the online tools listed in Table S4. Vector NTI version 11.5 and MEGA version 5.1 were used for multiple sequence alignments and evolutionary tree production, respectively. The structural domain of proteins was predicted by the online tools listed in Table S4 and consultation of reference domains in homologous MFN2 and DRP1 proteins. The amino acid sequences of the homologous proteins were downloaded from the National Center for Biotechnology Information (https://www.ncbi.nlm.nih.gov/ (accessed on 3 February 2022)), and its GenBank accession numbers are listed in Tables S5 and S6.

### 4.6. Quantitative Analysis of mfn2 and drp1 mRNA Expression

Both RNA extraction from the coelomic fluid, body wall, intestine, retractor muscle, and nephridium using TRIzol (Cwbio) and reverse transcription using the PrimeScript^®^ RT Reagent Kit (Takara) were performed according to the manufacturers’ instructions. The primers for qPCR were designed by Primer Premier 5.0 software and are listed in Tables S2 and S3. *gapdh* was used as the internal control. For PCR, a 20 μL reaction volume was setup and contained 10 μL of Master Mix, 3 μL of PCR-grade water, 5 μL of 1:20 diluted cDNA, and 1 μL of each primer (10 μM). The Roche LightCycler480 System was used for the qPCR assay, and the PCR procedure was as follows: 95 °C for 3 min, followed by 40 cycles of 95 °C for 10 s, 55 °C for 15 s, and 72 °C for 15 s. The comparative delta–delta Ct method was used for analyzing the relative gene expression level. Five to six samples were used for each experiment to analyze the mRNA expression.

### 4.7. Western Blotting

RIPA lysis buffer (Beyotime) was used according to the manufacturer’s instructions to extract the total protein from coelomic fluid. The protein concentration was measured using a BCA protein assay kit (Beyotime). The protein samples were then mixed with SDS-PAGE loading buffer (Beyotime) and denaturized at 100 °C for 8 min. The samples were separated on 4–15% SDS-PAGE gels and transferred to PVDF membranes. The membranes were blocked with 5% skimmed milk in 0.1% Tween 20 in TBS buffer (TBST) for 2 h, incubated in primary antibody in TBST (1:500 dilution) overnight at 4 °C, washed 3 times in TBST for 36 min, incubated in secondary antibody in TBST (horseradish peroxidase-labeled goat anti-rat IgG [H+L], Beyotime; 1:2000 dilution) for 2 h at 37 °C, and washed 3 times in TBST for 45 min. Finally, the membranes were incubated with developer and photographed using a chemiluminescence imaging system. The gray scale values of MFN2, DRP1 and ACTB proteins blotting were measured by using ImageJ software (National Institutes of Health, Baltimore, MD, USA). The ratio of their gray scale values were used to analyze the relative expression of the MFN2 and DRP1 proteins. Three samples were used for each experiment.

### 4.8. Statistical Analysis

Experimental data were expressed as means ± standard deviation. All statistics were performed using SPSS Software 21.0 (SPSS, Inc., Chicago, USA). For normally distributed datasets with homogeneity of variance, the significance of the differences was determined using one-way analysis of variance (ANOVA), followed by a post-hoc Tukey’s multiple comparison test. If not normally distributed or heteroscedasticity, non-parametric tests were chosen to compare the significance of the means. *p* < 0.05 was considered significant. Data for statistical analysis in Figure 2, Figure 3, Figure 4 and Figure 5 are shown in Tables S7–S10.

## 5. Conclusions

Mitochondria localized near the nucleus during spermiogenesis. Their major axis length, minor axis length, average diameter, eccentricity, cross-sectional area, and volume changed dramatically, indicating the occurrence of mitochondrial fusion and fission during spermiogenesis. Mitochondrial dynamics-related proteins MFN2 and DRP1 are conserved with high homology in animals. The expression levels of *Pe*-MFN2 and *Pe*-DRP1 were highest in coelomic fluid and first increased and then decreased from March to December. The expression in coelomic fluid during the breeding season was significantly higher than that during the non-breeding season. In addition, *Pe*-MFN2 and *Pe*-DRP1 are mainly found in spermatids in coelomic fluid and are consistently expressed during spermiogenesis. The morphological features of mitochondria and the expression patterns of MFN2 and DRP1 suggest that mitochondrial dynamics plays a role in spermiogenesis and may regulate mitochondrial physiological function to promote or adapt spermatid development. The results provide valuable information for exploring the molecular mechanisms of spermatogenesis in *P. esculenta* and can be used as a theoretical reference for promoting male germ cell maturation.

## Data Availability

The authors declare that all the data supporting the findings of this study are available within the article.

## Supplementary Materials

The following supporting information can be downloaded at: https://www.mdpi.com/article/10.3390/ijms232415517/s1.
