# Supplementary material for: Mitochondrial Features and Expressions of MFN2 and DRP1 during Spermiogenesis in Phascolosoma esculenta
Source: Int J Mol Sci. 2022 Dec 8;23(24):15517. doi: 10.3390/ijms232415517 (PMC9778712; doi:10.3390/ijms232415517)
Supplement: Supplementary file 1 [file ijms-23-15517-s001.zip › Supplementary File-Figures.pdf]

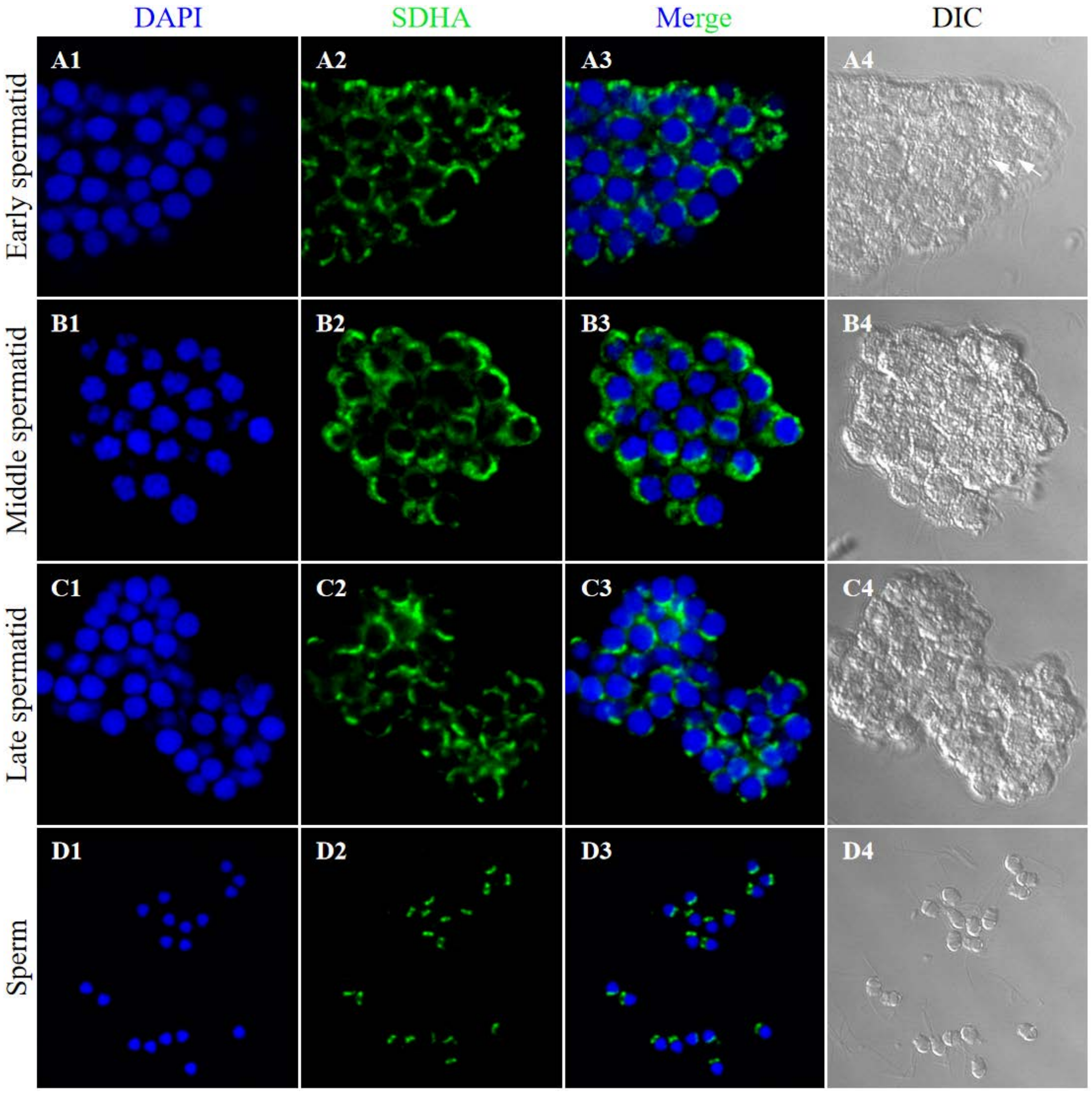

Figure S1 Distribution of mitochondria during spermiogenesis. (A1–A4) Early spermatid; (B1–B4) middle spermatid; (C1–C4) late spermatid; (D1–D4) sperm. Blue indicates nuclei stained with DAPI; green indicates SDHA, a mitochondrial marker. DIC: Differential interference contrast imaging.

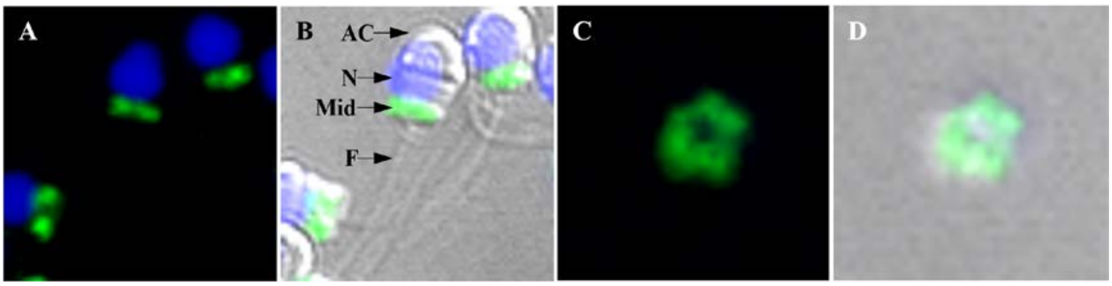

Figure S2 Microstructure of sperm and cross-section of midpiece. (A) Nuclei and mitochondria of sperm. (B) Differential interference contrast (DIC) imaging of **A**, showing the microstructure of sperm. (C) Vertical section of the sperm midpiece, showing the mitochondria. (D) DIC imaging of **C**. AC: acrosome, F: flagellum, Mid: midpiece, N: nucleus.

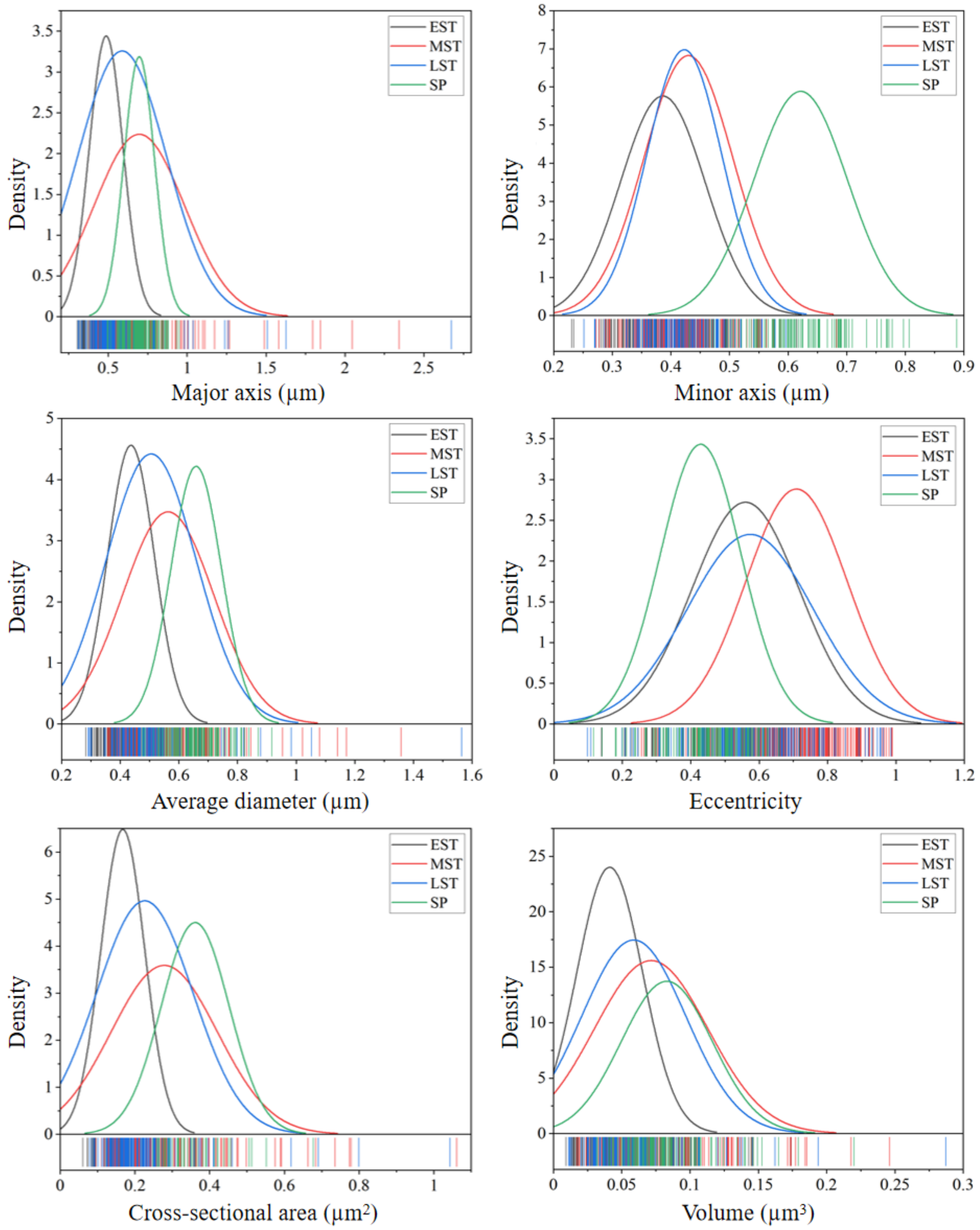

Figure S3 Distribution curves of mitochondrial long axis length, short axis length, diameter, eccentricity, cross-sectional area, and volume during spermiogenesis. EST: early spermatid, MST: middle spermatid, LST: late spermatid, SP: sperm

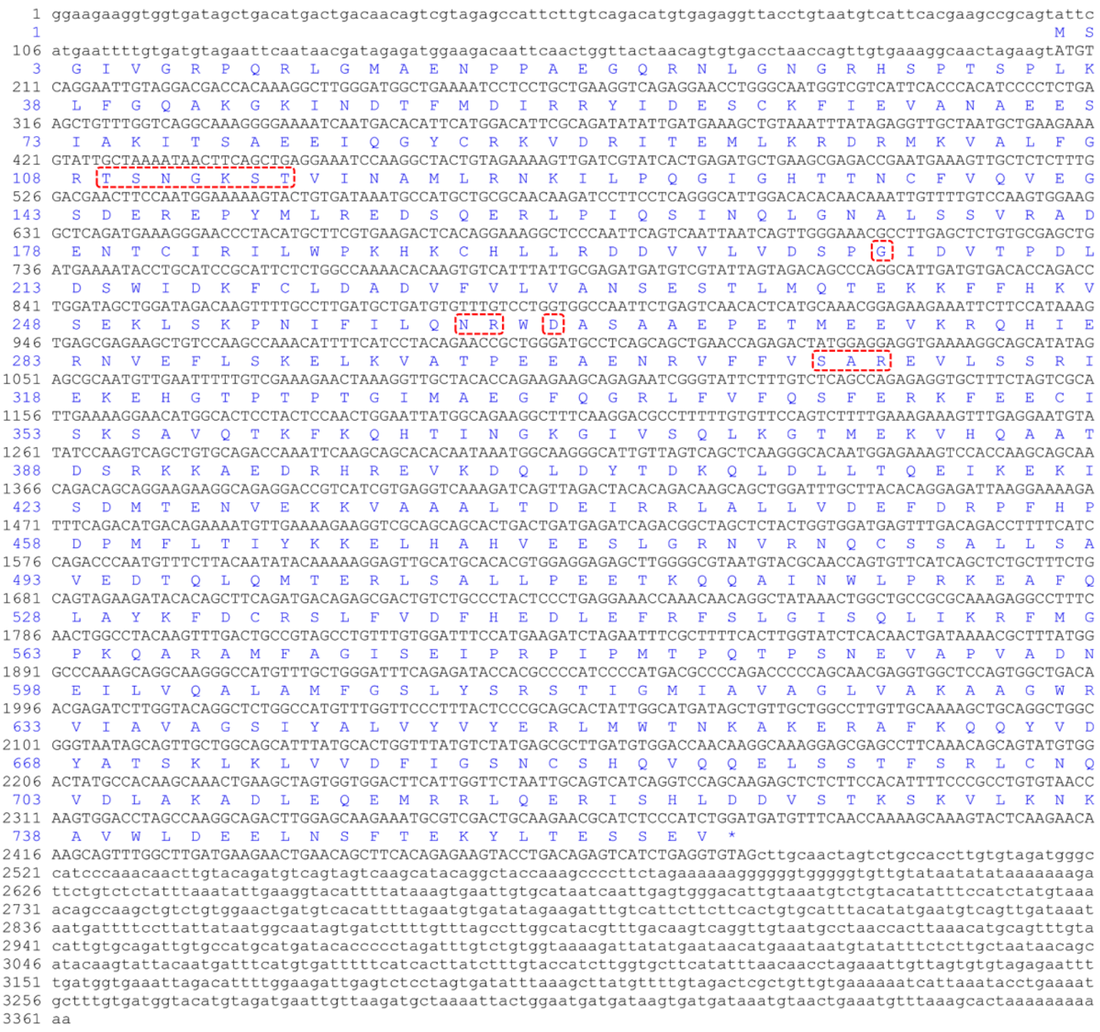

Figure S4 Full-length cDNA of *Pe-mfn2*. The full length of *Pe-mfn2* cDNA is 3362 bp, consisting of a 206-bp 5′ untranslated region, a 879-bp 3′ untranslated region, and a 2277-bp open reading frame encoding 759 amino acids. Lowercase letters represent the untranslated region; uppercase letters represent the open reading frame. The blue letters above the base sequence represent the corresponding amino acid sequence. The initiation codon is ATG, and the termination codon is TAG. The red boxes denote GTP binding sites.

```
   1                                                                                            M  E  N  L  I
   1 gaaatgaaaatcgccatatctgacaaggagagatcatacaaaatttaacggttaaatgtgacagagtatttcataggaaaaaaattgttcgATGGAGAATTTGAT
   6  P  V  I  N  K  L  Q  D  V  F  N  T  V  G  S  E  S  V  Q  L  P  Q  I  I  V  I  G  T  Q  S  S  G  K  S  S
 106 CCCTGTGATTAACAAGCTACAAGATGTGTTCAACACTGTTGGATCTGAATCTGTTCAATTGCCCCAAATCATAGTAATTGGTACACAGAGCAGTGGGAAAAGTTC
  41  V  L  E  S  L  V  G  R  D  F  L  P  R  G  T  G  I  V  T  R  R  P  L  V  L  Q  L  V  Y  V  P  A  D  D  K
 211 GGTTTTGGAGAGCCTGGTAGGACGAGACTTCCTGCCACGTGGAACAGGCATCGTCACCAGGCGACCACTGGTGCTCCAGCTAGTCTATGTGCCAGCTGATGATAA
  76  E  T  R  L  Q  E  S  G  G  R  G  D  S  I  Q  A  E  E  W  A  K  F  L  H  T  K  N  K  I  Y  T  D  F  N  E
 316 GGAAACCAGGCTACAAGAGAGTGGAGGCAGGGGTGATAGCATTCAAGCTGAAGAATGGGCCAAGTTCCTTCACACAAAGAACAAAATATATACAGACTTCAACGA
 111  I  R  R  E  I  D  N  E  T  D  R  L  S  G  S  N  K  G  I  C  P  D  P  I  S  L  K  I  F  S  P  R  V  L  N
 421 AATCAGGAGGGAGATTGACAATGAGACAGATCGCCTTTCAGGTTCTAATAAGGGGATCTGTCCAGATCCAATTAGCTTGAAGATCTTTTCTCCACGGGTGCTGAA
 146  L  T  L  V  D  L  P  G  L  T  K  V  P  V  G  D  Q  P  E  D  I  E  H  Q  I  R  D  M  C  L  Y  Y  I  S  N
 526 CCTGACCCTCGTTGATCTCCCAGGGCTGACCAAGGTACCAGTTGGTGACCAGCCAGAAGACATCGAGCACCAGATCAGGGACATGTGTCTATACTACATCTCCAA
 181  P  N  S  I  I  L  A  V  T  A  A  N  T  D  M  A  T  S  E  A  L  K  L  A  K  E  V  D  P  D  G  R  R  T  L
 631 CCCCAACTCTATCATTCTGGCTGTCACCGCTGCCAACACCGACATGGCCACCTCCGAGGCACTCAAGCTGGCAAAGGAGGTCGACCCGGATGGGAGGAGAACACT
 216  A  V  V  T  K  L  D  L  M  D  Q  G  T  D  A  M  E  V  L  C  G  R  V  I  P  V  K  L  G  I  I  G  V  V  N
 736 GGCTGTGGTCACTAAACTTGACCTGATGGACCAAGGCACAGATGCCATGGAGGTGCTGTGTGGGAGGGTCATTCCTGTCAAGCTGGGAATCATCGGCGTGGTCAA
 251  R  S  Q  A  D  I  N  S  N  K  E  I  S  D  A  L  K  N  E  A  S  F  L  Q  K  K  Y  P  G  I  A  N  R  N  G
 841 CCGGAGTCAGGCTGACATCAACTCAAACAAGGAAATATCAGATGCCTTGAAAAATGAGGCATCTTTCCTACAGAAGAAGTATCCTGGCATCGCCAACAGAAACGG
 286  T  P  Y  L  A  R  T  L  N  R  L  L  M  H  H  I  R  D  C  L  P  E  L  K  T  R  V  N  V  M  A  A  Q  F  Q
 946 CACGCCCTACCTGGCCAGGACACTCAATAGGTTGCTGATGCACCATATCCGAGACTGTTTGCCGGAACTGAAGACGAGGGTGAATGTGATGGCAGCTCAGTTTCA
 321  Q  L  L  N  S  F  G  D  E  I  T  D  K  G  Q  V  L  L  Q  M  I  T  K  F  A  T  A  Y  C  N  T  I  E  G  T
1051 GCAGCTGCTCAACTCATTTGGTGATGAGATCACAGACAAGGGTCAGGTTCTGCTACAAATGATCACAAAGTTTGCAACTGCCTACTGTAATACAATAGAAGGAAC
 356  P  K  N  I  E  T  T  E  L  C  G  G  A  R  I  C  Y  I  F  H  E  T  F  G  K  T  L  E  S  V  A  A  L  G  G
1156 TCCAAAGAATATTGAAACCACAGAATTGTGTGGTGGTGCTAGAATCTGCTATATCTTCCATGAAACCTTTGGGAAAACCTTAGAAAGTGTTGCTGCCCTCGGAGG
 391  L  S  T  L  D  V  L  T  A  I  R  N  A  T  G  P  R  P  A  L  F  V  P  E  V  S  F  E  L  L  V  K  R  Q  I
1261 CTTGTCTACATTAGATGTTCTCACAGCTATACGCAATGCCACGGGTCCAAGGCCTGCTTTGTTTGTACCTGAAGTCTCATTTGAGTTGCTGGTGAAGAGGCAGAT
 426  R  R  L  E  E  P  S  L  R  C  V  E  L  V  H  E  E  M  Q  R  I  I  Q  H  C  G  T  Q  Q  E  M  M  R  F  P
1366 CCGGAGATTAGAGGAGCCCAGTCTCAGATGTGTGGAGTTGGTGCACGAGGAGATGCAGAGGATCATACAGCACTGTGGAACACAGCAAGAAATGATGAGATTCCC
 461  K  L  H  E  R  I  V  D  V  V  T  A  L  L  R  R  R  L  P  I  T  N  D  M  V  T  N  L  V  A  I  E  L  A  Y
1471 AAAACTGCATGAGAGAATTGTTGATGTGGTAACAGCGCTACTACGGCGACGGCTGCCCATCACCAATGACATGGTGACCAACCTAGTAGCCATTGAGCTTGCATA
 496  I  N  T  K  H  P  D  F  K  E  A  G  L  V  H  K  A  L  T  E  N  L  D  H  E  M  R  K  M  A  M  E  R  D  H
1576 CATCAATACCAAACACCCGGACTTCAAGGAGGCGGGGCTGGTGCACAAGGCACTGACAGAGAACCTGGACCACGAGATGAGGAAGATGGCGATGGAACGAGATCA
 531  M  I  R  A  P  P  Q  E  D  K  N  S  S  L  S  P  I  Q  N  K  P  V  Q  N  N  I  N  T  S  S  W  M  A  N  F
1681 CATGATCAGGGCTCCCCCACAGGAAGACAAGAATAGCTCCCTCAGCCCAATACAAAACAAACCAGTTCAGAACAACATCAACACATCCTCATGGATGGCCAACTT
 566  M  K  T  G  K  I  E  P  A  E  G  R  P  G  S  P  S  S  S  G  S  V  P  T  S  T  E  P  S  P  S  R  T  K  S
1786 CATGAAGACGGGTAAGATTGAACCGGCTGAAGGGCGGCCGGGCAGCCCGAGTAGTTCAGGCAGTGTACCCACCAGCACAGAACCATCACCTTCCAGGACCAAGTC
 601  T  G  V  N  L  L  D  E  V  P  A  V  P  S  S  R  K  L  S  S  R  E  Q  R  D  C  D  V  I  E  R  L  I  R  S
1891 AACAGGCGTCAACCTTCTCGATGAAGTGCCAGCTGTACCCTCAAGTAGAAAACTGTCTTCAAGAGAACAAAGAGACTGTGACGTTATAGAGCGGTTAATAAGGTC
 636  Y  F  L  I  V  R  K  N  I  Q  D  S  V  P  K  A  I  M  H  F  L  V  N  H  I  K  E  H  L  Q  S  E  L  V  S
1996 GTACTTCCTAATCGTGAGGAAAAACATCCAAGACAGTGTACCGAAGGCTATCATGCACTTCCTGGTCAACCACATCAAGGAACATCTGCAAAGCGAACTAGTCAG
 671  H  L  Y  K  E  K  E  F  D  K  L  L  E  E  S  E  N  I  A  A  R  R  R  E  A  A  D  M  L  Q  A  L  Q  K  A
2101 TCACCTGTACAAGGAGAAAGAATTCGACAAACTACTAGAAGAGTCAGAGAACATCGCGGCCAGGAGACGGGAGGCAGCTGATATGCTACAGGCGCTACAAAAAGC
 706  S  Q  I  I  S  E  I  R  E  T  H  V  W  *
2206 CAGCCAAATAATCAGTGAAATCAGAGAAACACACGTATGGTGAaacaagtagaagctaggtcctttgtatgagagttgtatgtggatgatgtacataatgtgtag
2311 gcgccacaatatgccagtgtgataggcattcatacagtttgcaaggaatgtccatataacagaggtggaagttatccagaaattaccagaattttcggatatttc
2416 tctagctttctgaacatgttatgatttaatattataaaaaatacagaggtaaaaatgataaaaactaccttaatatacaatcctttatagcgtacatttatcatg
2521 caaacaaattaccttccttttggcttctgtggcattagaacatgcagaacgatattttgacagcatgaattgggagattatatcaccagagaccccaagctttt
2626 taattttgacagcataataaccatttcagtttttggagctgggacaaaaagttccatttttttggccccttgccacttgcgcccctgatatatagaacttcatacc
2731 tactgcgtggagagtccttaaggactgcatctgaaagttatcccccaacctgttgtcagtagtttgcatcataatacaataagagagagattaactatatatcag
2836 gtgtgacccaataaagttgtgtgtcattctgtacatagagacagatgtatttgatttagcctcttcatgtcaacttgcttaattgctaaaggagatctacgtaca
2941 attgttaaataatgcacaactttgtgttgctatagcaactacatttctgtgtttgtagtatatgaaattagctgtacacatcacaggtacaaaatagtttttact
3046 actgaaattaatgtaagaaatatgtttgtagtgccatgtttcttgtcttctttttgtgatgcctattgtgttctttttttactaggaaactttacccttcatgtct
3151 gtgaagcaaccaacctaggagtaacatatgtactatggagggcagaaagaatgaacgaagtgtgaggttgtatttagtttattagtttcagcagtctggttgaag
3256 ttggaattgcaacagggttgtcacctgtttaatgtgtgcatgatagtatcacatgtcagataatataatttttttctgtataggggagagatatgattttgagtt
3361 ttaacttattagactcttttaaatttagcattatcatatatagaactggtaataacgttcccattcagtatgatattatcaggttaacaagggtttcagtaagtt
3466 tgaattggccggctattggctgaaagtaggtggctaaaactggtgcaagttgtaaaaacatcgtggtgtcaaaagtagccaccttcacaggcataagaccaagaa
3571 aaaaaaaacatgtttctggttggcgtgctggtcatattaaaatccaaaaaaaaaaaaaaaaaaaaaaaaaa
```

Figure S5 Full-length cDNA of *Pe-drp1*. The full length of *Pe-drp1* cDNA is 3643 bp, consisting of a 91-bp 5′ untranslated region, a 1398-bp 3′ untranslated region, and a 2154-bp open reading frame encoding 718 amino acids. Lowercase letters represent the untranslated region; uppercase letters represent the open reading frame. The blue letters above the base sequence represent the corresponding amino acid sequence. The initiation codon is ATG, and the termination codon is TGA. The red boxes denote GTP binding sites.

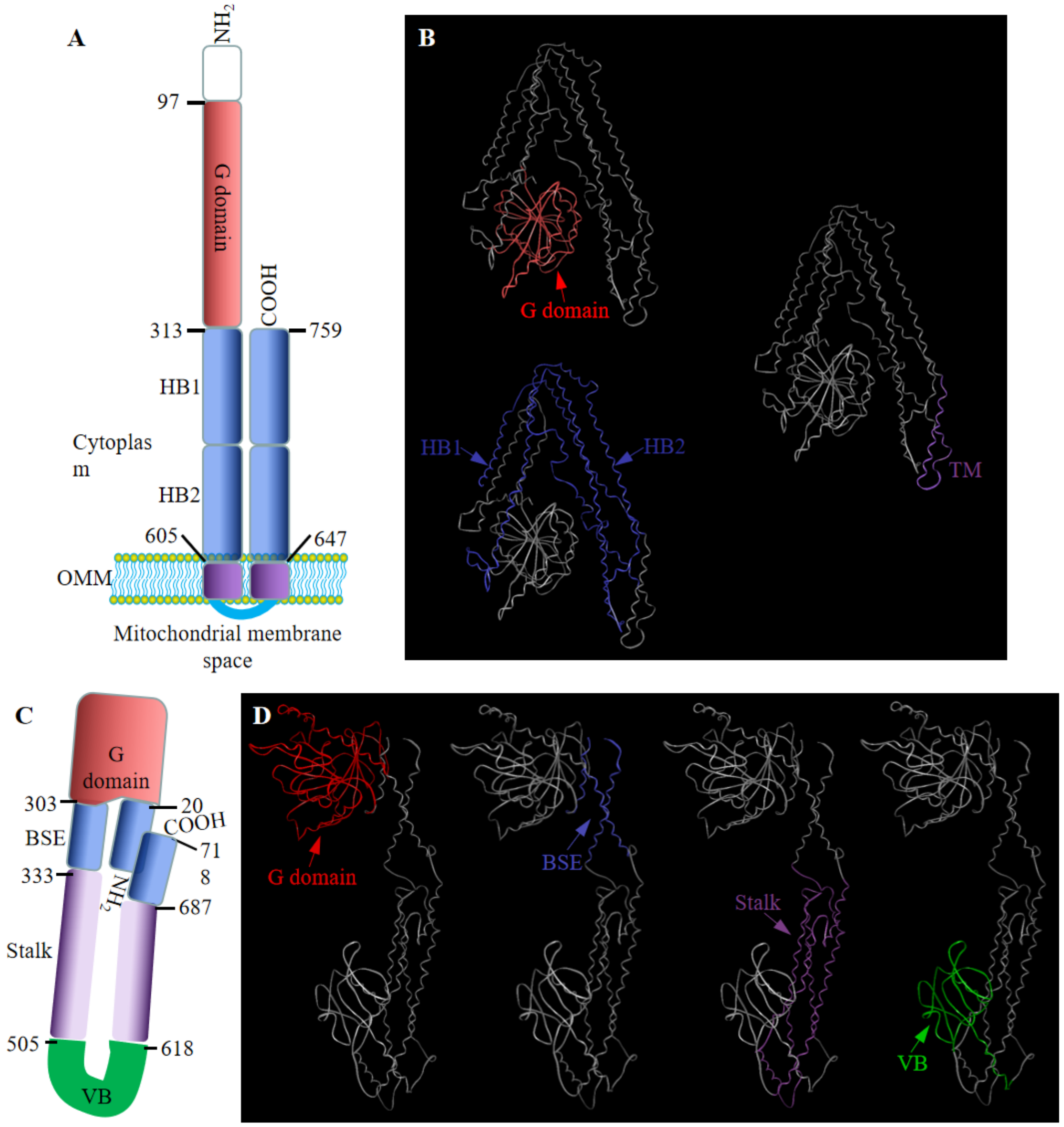

Figure S6 Domains and 3D structures of *Pe*-MFN2 and *Pe*-DRP1. (A) Diagram of *Pe*-MFN2 structure domain. (B) The predicted tertiary structure of *Pe*-MFN2. (C) Diagram of *Pe*-DRP1 structure domain. (D) The predicted tertiary structure of *Pe*-DRP1. G domain: GTPase domain; HB: helical bundle regions; OMM: outer mitochondrial membrane; TM: transmembrane domain; BSE: bundle signaling element; VB: variable domain

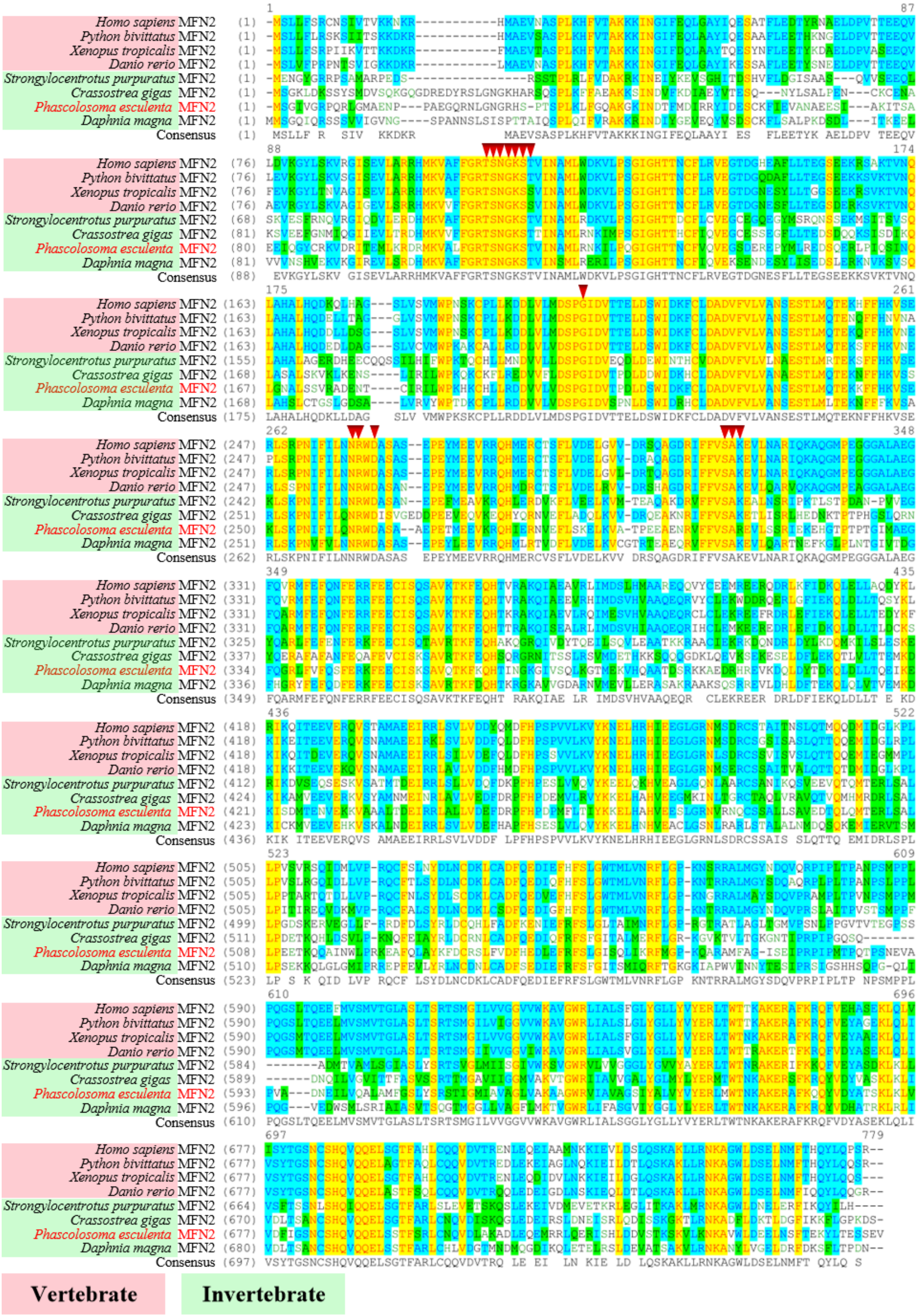

Figure S7 Comparison of MFN2 homologous proteins. Identical amino acids are highlighted in yellow. The bluish green regions demonstrate at least 50% similarity, and the green regions have less similarity. The red triangles indicate GTP binding sites. The consensus positions and identity positions of *Pe*-MFN2 with MFN2 in *Mus musculus*,

Python bivittatus, *Xenopus tropicalis*, *Danio rerio*, *Strongylocentrotus purpuratus*, *Crassostrea gigas*, and *Daphnia magna* are respectively 65.7% and 49.9%, 64.4% and 49.2%, 66.1% and 50.8%, 65.6% and 51.59%, 63.7 % and 48.49%, 65.4% and 53.19%, and 65.4% and 50.29%.

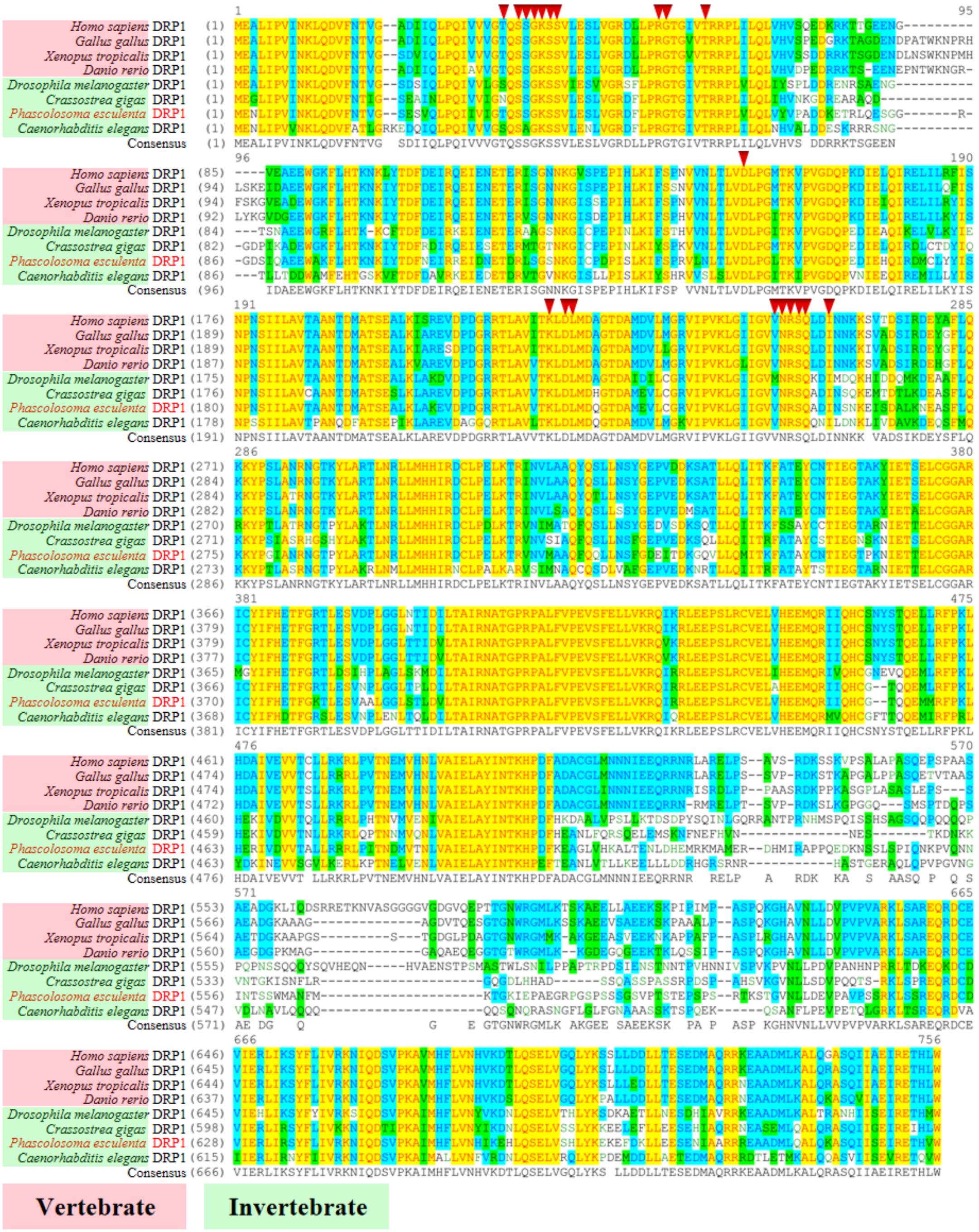

Figure S18 Comparison of DRP1 homologous proteins. Identical amino acids are highlighted in yellow. The bluish green regions demonstrate at least 50% similarity, and the green regions have less similarity. The red triangles indicate GTP binding sites. The consensus positions and identity positions of *Pe*-DRP1 with DRP1 in *Homo sapiens*, *Gallus gallus*, *Xenopus laevis*, *Danio rerio*, *Drosophila melanogaster*, *Crassostrea gigas*, and *Caenorhabditis elegans* are respectively 78.5% and 67.6%, 78.1% and 67.4%, 77.7% and 67.3%, 78.9% and 68.0%, 78.1% and 66.5%, 80.8% and 71.9%, and 72.9% and 61.2%.

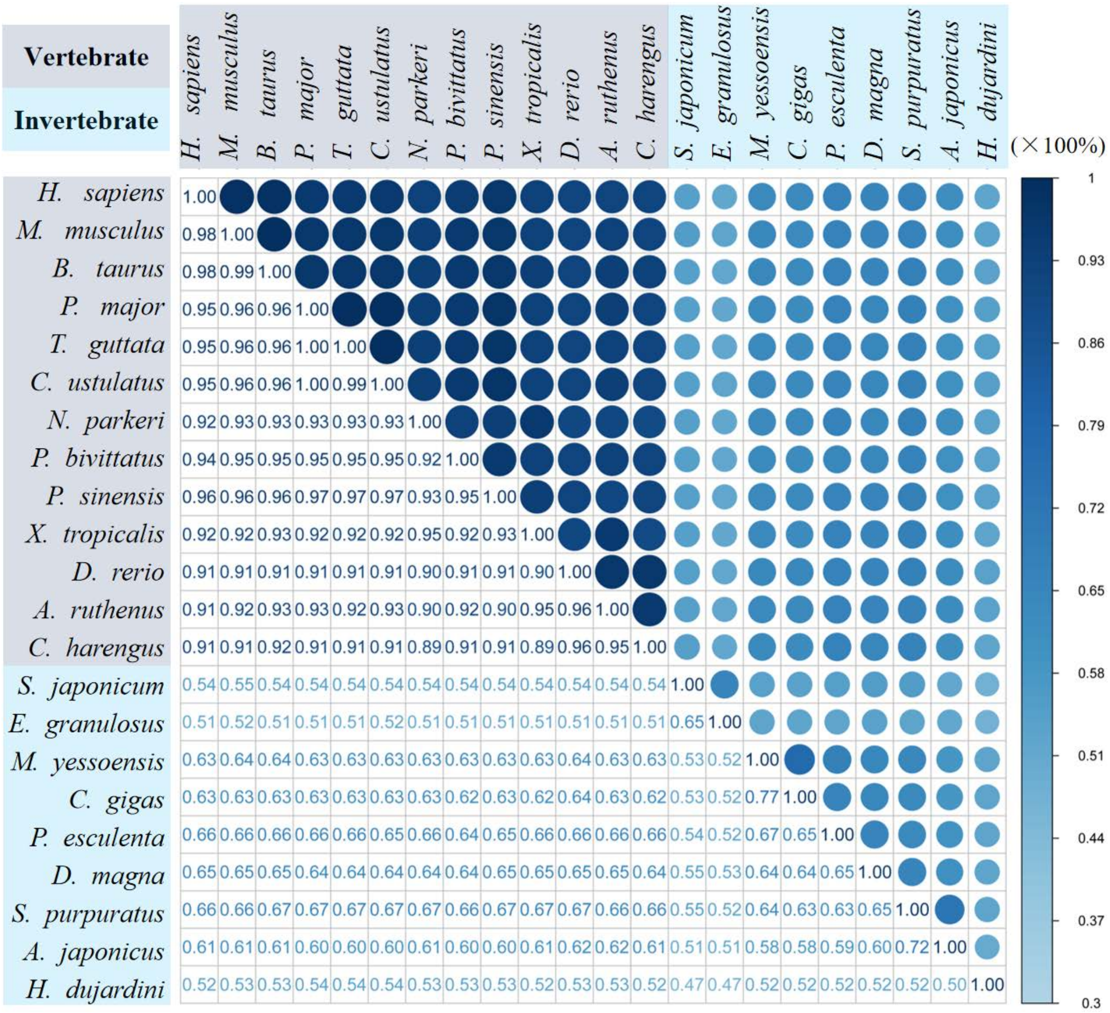

Figure S9 Consensus positions between MFN2 homologous proteins.

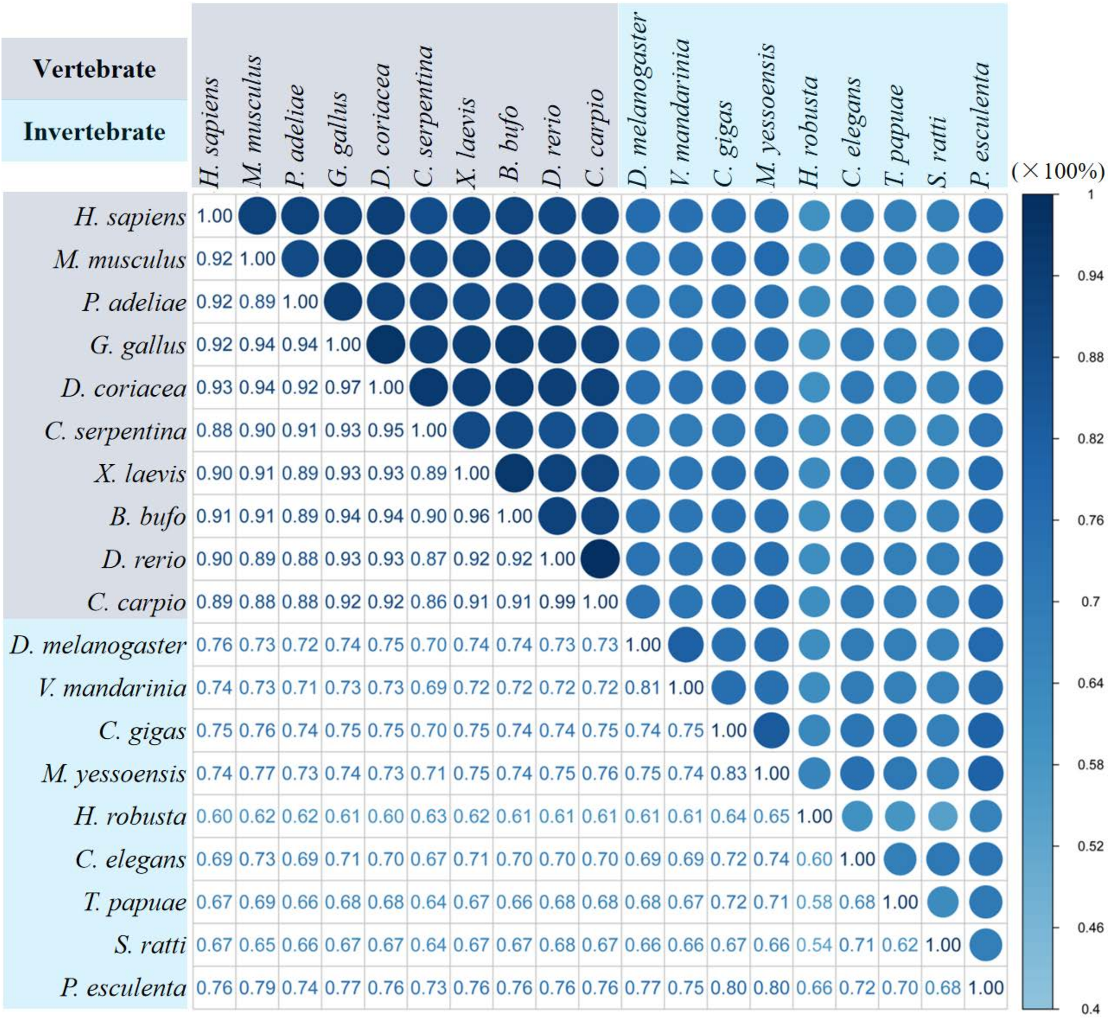

Figure S10 Consensus positions between DRP1 homologous proteins.

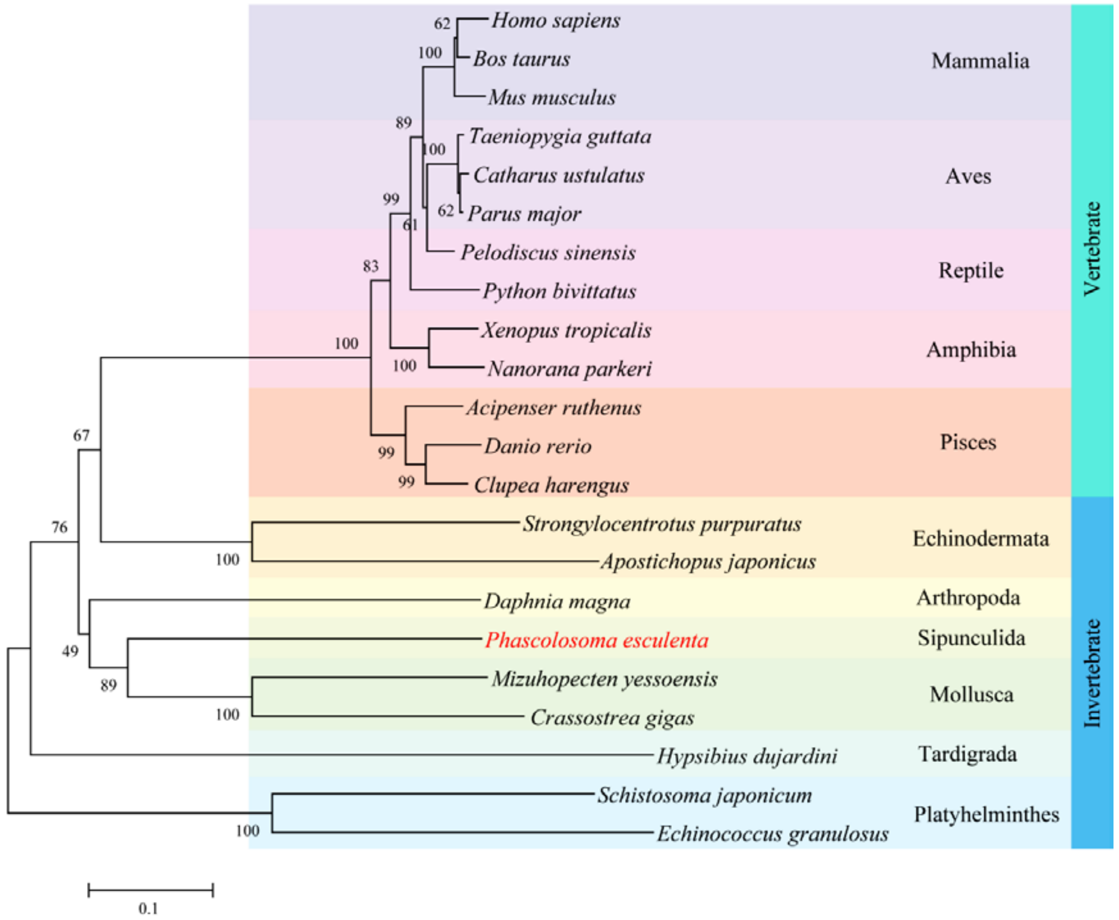

Figure S11 Phylogenetic tree based on the amino acid sequence of MFN2. MEGA version 5.1 software was used to construct the neighbor-joining phylogenetic tree. *Phascolosoma esculenta* is marked in red font. Among the selected proteins homologous to MFN2, *Pe*-MFN2 is most closely related to mollusk MFN2.

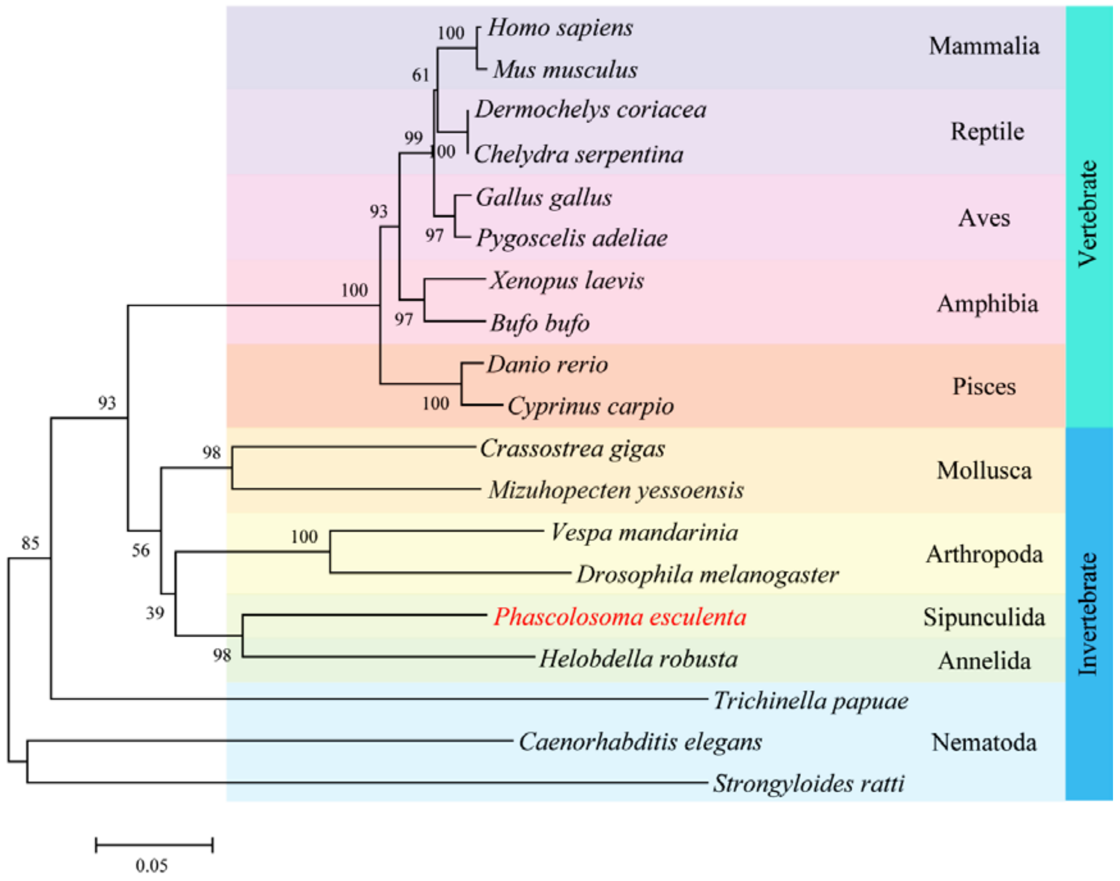

Figure S12 Phylogenetic tree based on the amino acid sequence of DRP1. MEGA version 5.1 software was used to construct the neighbor-joining phylogenetic tree. *Phascolosoma esculenta* is marked in red font. Among the selected proteins homologous to DRP1, *Pe*-DRP1 is most closely related to Annelida DRP1.

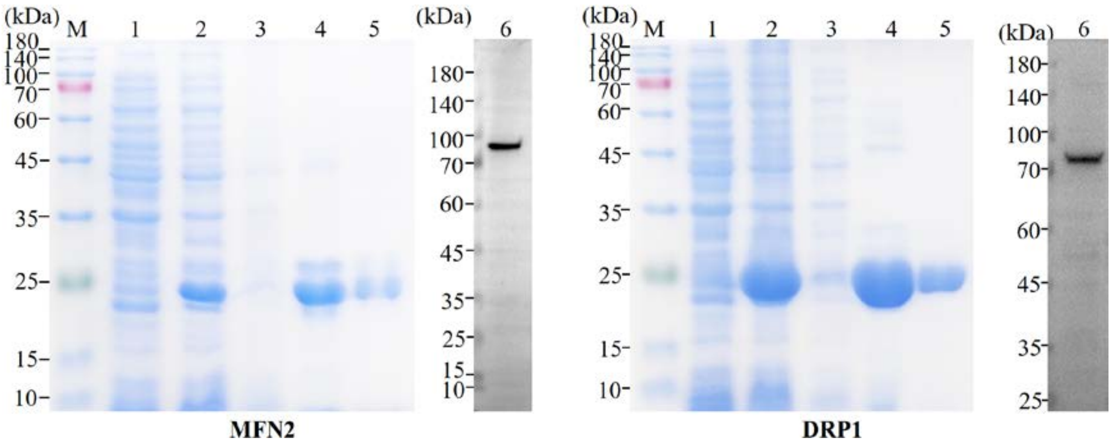

Figure S13 Expression and purification of recombinant proteins and specific analysis of rat anti-*Pe*-MFN2 and rat anti-*Pe*-DRP1 antibodies. Line 1 shows the total protein of DE3-MFN2 or DE3-DRP1 *Escherichia coli* [Transetta (DE3) *E. coli* that can express the MFN2 or DRP1 recombinant protein] without isopropylthio-β-galactoside (IPTG) induction. Line 2 shows the total protein of DE3-MFN2 or DE3-DRP1 *E. coli* induced by IPTG. Line 3 shows the soluble protein of DE3-MFN2 or DE3-DRP1 *E. coli* induced by IPTG. Line 4 shows the insoluble protein of DE3-MFN2 or DE3-DRP1 *E. coli* induced by IPTG. Line 5 shows the purified recombination proteins. Line 6 shows western blot analysis using the rat anti-*Pe*-MFN2/DRP1 antibody. Protein bands were detected at approximately 86 kDa and 80.5 kDa, consistent with the predicted molecular weights of MFN2 and DRP1, respectively, indicating that the produced antibody has high specificity.

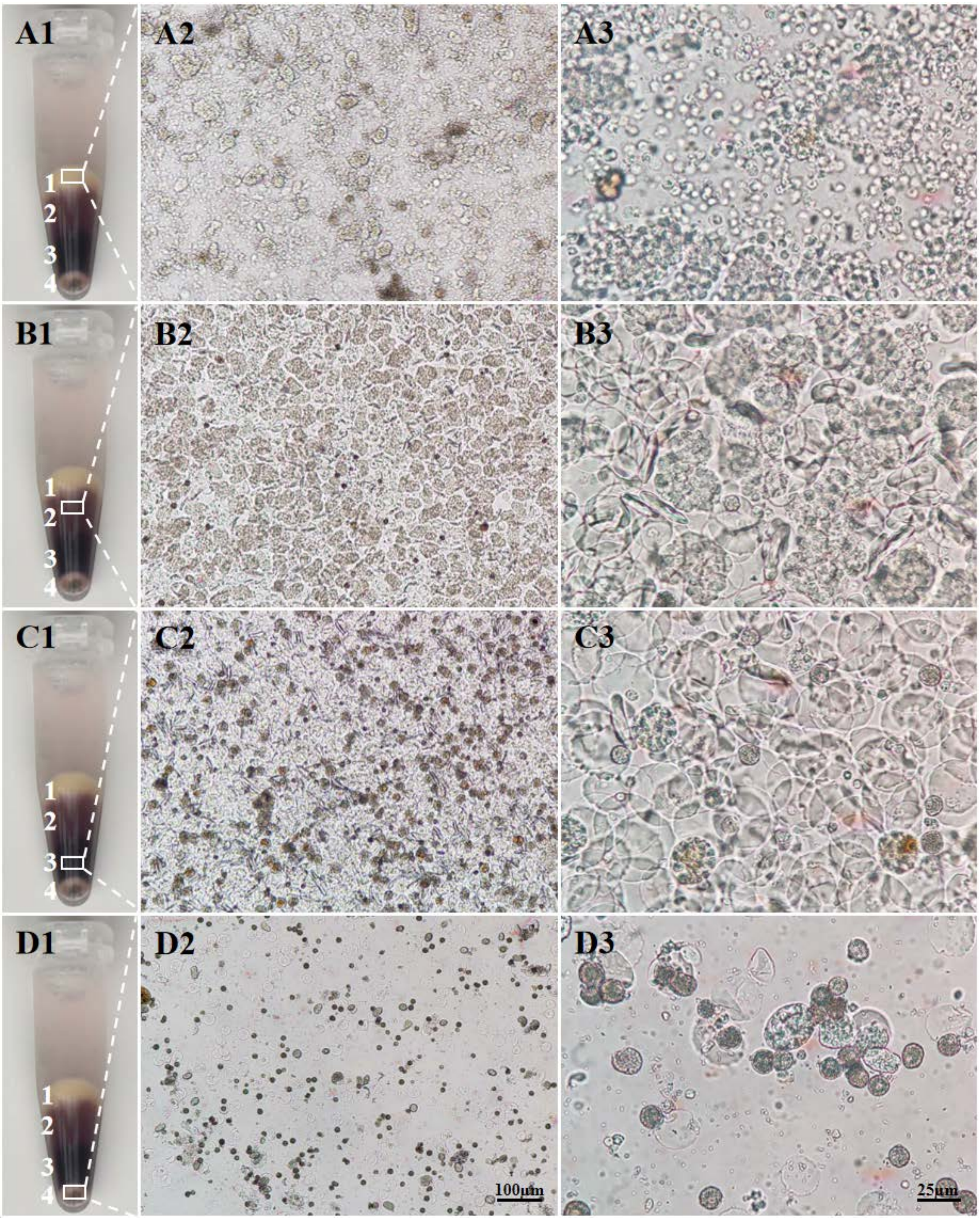

Figure S14 Separation of components in coelomic fluid by centrifugation. (A1–A3) Layer 1; (B1–B3) Layer 2; (C1–C3) Layer 3; (D1–D3) Layer 4. The top layer is composed of spermatids. Layer 2 is mainly composed of spermatids and a very small number of blood cells. Layer 3 is mainly composed of blood cells and granule cells. Layer 4 is mainly composed of granule cells.

```
   1 M  A  T  L  L  K  A  V  S  T  R  I  V  P  K  A  A  S  T  N  V  V  N  T  L  T  K  P  L  G
   1 ATGGCCACCCTCCTGAAAGCGGTGTCAACGCGGATAGTGCCAAAAGCCGCATCAACTAACGTGGTGAACACATTAACAAAACCTTTGGGA
  31 A  T  R  D  F  S  F  R  V  H  G  K  N  A  Q  A  G  V  S  K  T  E  Q  I  S  R  D  Y  P  V
  91 GCCACAAGAGATTTTTCATTCAGAGTACATGGCAAAAATGCACAGGCAGGTGTCAGCAAAACTGAACAGATCTCAAGAGACTACCCCGTA
  61 V  D  H  T  Y  D  A  V  V  V  G  A  G  G  A  G  L  R  A  A  F  G  L  A  Q  D  G  F  K  T
 181 GTCGACCACACATATGATGCTGTGGTAGTTGGAGCAGGAGGGGCTGGTCTTCGAGCTGCCTTCGGATTGGCCCAGGATGGCTTTAAAACA
  91 A  C  I  T  K  L  F  P  T  R  S  H  T  V  A  A  Q  G  G  I  N  A  A  L  G  N  M  E  E  D
 271 GCATGCATCACAAAACTCTTCCCAACACGCTCCCACACTGTTGCTGCCCAGGGAGGGATCAATGCAGCCCTGGGTAACATGGAGGAGGAT
 121 D  W  R  Y  H  F  Y  D  T  V  K  G  S  D  W  L  G  D  Q  D  A  I  H  Y  M  T  E  Q  A  P
 361 GACTGGAGGTACCATTTCTATGACACAGTCAAGGGATCTGATTGGCTGGGAGACCAGGATGCGATTCATTATATGACTGAGCAGGCACCA
 151 Q  A  V  I  E  L  E  N  Y  G  M  P  F  S  R  L  E  N  G  K  I  Y  Q  R  A  F  G  G  Q  S
 451 CAGGCAGTCATTGAGCTAGAAAACTATGGCATGCCCTTCAGCCGTCTGGAAAATGGCAAAATCTACCAGCGCGCATTCGGAGGACAGAGC
 181 L  K  F  G  T  G  G  Q  A  H  R  C  C  C  V  A  D  R  T  G  H  S  M  L  H  T  L  Y  G  R
 541 TTGAAGTTTGGCACAGGAGGCCAAGCCCACAGATGCTGCTGTGTTGCTGACAGGACCGGTCACTCTATGTTGCACACTCTCTATGGAAGG
 211 S  L  Y  Y  D  T  N  Y  F  I  E  Y  F  A  L  D  L  I  M  E  G  G  E  C  R  G  V  V  A  L
 631 TCACTGTATTATGACACCAACTACTTCATTGAGTACTTTGCCCTGGATCTTATCATGGAGGGTGGAGAGTGTCGTGGAGTGGTGGCCCTC
 241 C  L  E  D  G  S  I  H  R  F  H  A  K  N  T  V  L  A  T  G  G  Y  G  R  T  Y  F  S  C  T
 721 TGCCTGGAAGATGGCTCCATCCATCGCTTCCATGCTAAAAACACAGTGCTGGCCACAGGAGGTTACGGCAGAACATACTTCTCCTGCACA
 271 S  A  H  T  C  T  G  D  G  T  A  M  V  T  R  A  G  L  A  N  E  D  M  E  F  V  Q  F  H  P
 811 TCAGCACACACATGTACTGGGGATGGGACAGCCATGGTGACCAGAGCTGGCCTGGCAAATGAGGACATGGAGTTTGTCCAGTTCCACCCC
 301 T  G  I  Y  G  A  G  C  L  I  T  E  G  C  R  G  E  G  G  Y  L  L  N  S  E  G  E  R  Y  M
 901 ACAGGCATCTATGGAGCTGGCTGCCTGATCACAGAGGGCTGTAGGGGTGAGGGCGGCTATCTTCTGAACTCCGAAGGAGAGCGTTACATG
 331 E  R  Y  A  P  T  A  K  D  L  A  S  R  D  V  V  S  R  A  S  T  I  E  I  R  E  G  R  G  V
 991 GAGCGTTATGCTCCCACAGCCAAAGATCTGGCTTCTCGAGATGTGGTCTCCAGGGCAAGCACCATAGAAATTCGGGAGGGCAGAGGAGTG
 361 G  P  E  K  D  H  V  Y  L  Q  L  S  H  L  P  P  E  Q  L  Q  A  R  L  P  G  I  S  E  T  A
1081 GGTCCAGAGAAGGACCACGTATACCTACAGCTGTCCCATCTGCCCCCTGAACAGCTGCAGGCCCGCCTGCCAGGAATCTCAGAAACGGCC
 391 K  I  F  A  G  V  D  V  T  R  D  P  I  P  V  L  P  T  V  H  Y  N  M  G  G  V  P  T  N  Y
1171 AAGATCTTTGCTGGTGTTGACGTGACACGTGACCCCATCCCTGTGCTGCCCACCGTCCACTACAACATGGGTGGTGTTCCTACCAACTAC
 421 R  G  Q  V  I  Q  W  K  N  G  A  D  E  V  V  P  G  L  Y  A  C  G  E  A  A  C  A  S  V  H
1261 AGGGGACAGGTCATCCAGTGGAAGAACGGTGCTGATGAGGTGGTGCCAGGGCTGTATGCATGTGGCGAGGCTGCTTGTGCCTCAGTTCAT
 451 G  A  N  R  L  G  A  N  S  L  L  D  L  V  I  F  G  R  A  C  A  H  T  I  A  E  E  N  K  P
1351 GGTGCTAATAGGCTGGGCGCCAACTCACTCCTCGATCTGGTCATCTTTGGACGTGCCTGTGCACACACCATTGCTGAGGAAAACAAACCA
 481 G  D  T  I  G  E  I  S  R  D  A  G  E  S  S  V  A  N  M  D  K  L  R  H  A  N  G  N  I  K
1441 GGAGATACAATTGGTGAAATAAGCAGGGATGCCGGTGAATCGTCTGTTGCCAACATGGACAAACTGAGACATGCTAATGGTAACATCAAG
 511 T  A  E  L  R  L  N  M  Q  K  V  M  Q  T  H  A  A  V  F  R  D  G  Q  S  L  K  E  G  C  D
1531 ACAGCTGAACTCAGACTCAACATGCAGAAGGTGATGCAGACACATGCAGCGGTGTTTAGAGATGGACAGTCGCTGAAGGAGGGTTGTGAC
 541 K  M  D  A  I  Y  N  K  M  D  D  I  K  V  Y  D  R  G  L  V  W  N  S  D  L  V  E  T  L  E
1621 AAGATGGATGCTATCTACAACAAAATGGATGACATCAAGGTGTATGATCGAGGCCTGGTGTGGAACTCGGACCTTGTGGAGACGCTGGAG
 571 L  Q  N  L  M  L  N  A  M  Q  T  I  Y  S  A  E  A  R  K  E  S  R  G  A  H  S  R  E  D  F
1711 CTACAGAACCTGATGCTGAATGCAATGCAGACCATCTACAGTGCCGAGGCCAGGAAGGAGAGCCGAGGAGCCCACTCTCGCGAGGACTTT
 601 K  H  R  I  D  E  F  D  Y  S  K  P  L  E  G  Q  K  E  L  P  Y  D  Q  H  W  R  K  H  T  M
1801 AAGCACAGAATAGATGAATTTGACTACAGTAAACCACTGGAAGGTCAAAAAGAGTTGCCATACGACCAACACTGGAGAAAGCACACAATG
 631 S  Y  Q  D  V  N  S  G  K  V  T  L  D  Y  R  A  V  I  D  H  T  L  D  S  K  K  V  E  S  V
1891 TCCTACCAAGACGTAAACAGTGGAAAGGTTACATTAGACTATAGGGCTGTGATAGACCATACCTTAGACAGCAAAAAAGTGGAGTCTGTG
 661 P  P  K  I  R  S  Y  *
1981 CCACCAAAGATACGGTCATATTAA
```

Figure S15 Open reading frame of *Phascolosoma esculenta sdha* cDNA.

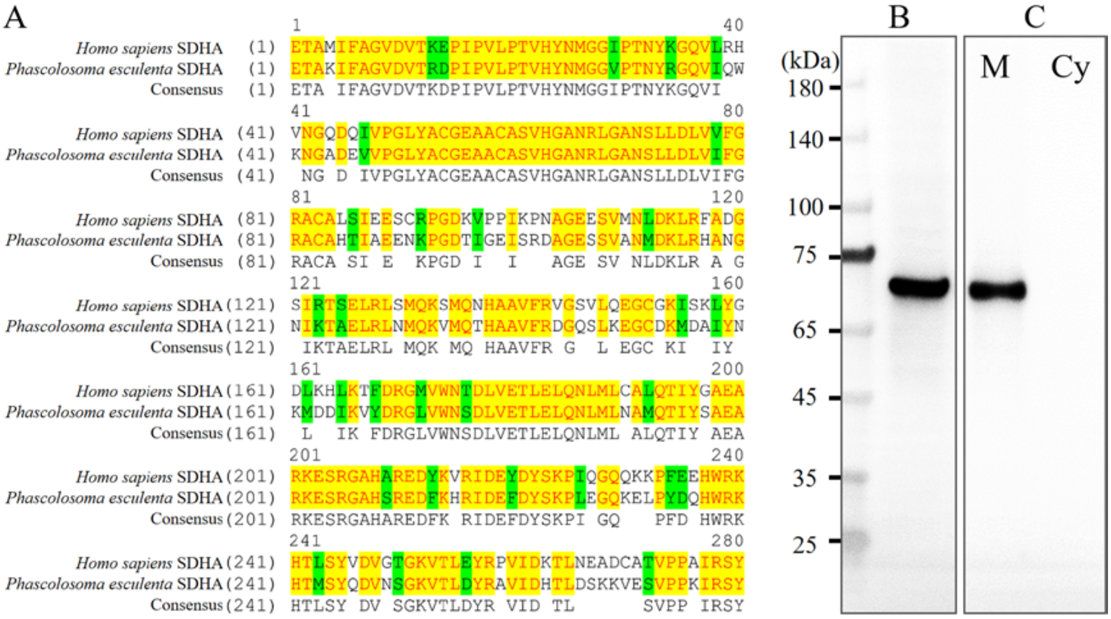

Figure S16 Comparison between antigen amino acid sequence of succinate dehydrogenase (SDHA) antibody and its homologous amino acid sequence in *Phascolosoma esculenta* SDHA, and specific analysis of SDHA antibody. (A) The sequence alignment of the antigen amino acids of the SDHA antibody and the homologous amino acids in *P. esculenta* SDHA; the consensus positions and identity positions were 80.4% and 69.3%, respectively. (B) SDHA was detected in the total protein of the coelomic fluid by western blotting (WB) using SDHA antibody; only a single protein band at approximately 73 kDa was detected. (C) SDHA in the total protein of mitochondria was detected by WB, but no SDHA was detected in the cytoplasm from which mitochondria were removed. Cy: total protein of cytoplasm from which mitochondria were removed, M: total protein of mitochondria.
