# Supplementary material for: Mitochondrial Features and Expressions of MFN2 and DRP1 during Spermiogenesis in Phascolosoma esculenta
Source: Int J Mol Sci. 2022 Dec 8;23(24):15517. doi: 10.3390/ijms232415517 (PMC9778712; doi:10.3390/ijms232415517)
Supplement: Supplementary file 1 [file ijms-23-15517-s001.zip › Supplementary File-Tables.pdf]

Table S1 Morphological parameters of mitochondria during spermiogenesis

Parameters	Early spermatid	Middle spermatid	Late spermatid	Sperm
Major axis (μm)	0.49 ± 0.11[c]	0.69 ± 0.29[a]	0.59 ± 0.28[b]	0.70 ± 0.10[a]
Minor axis (μm)	0.39 ± 0.07[c]	0.43 ± 0.09[b]	0.42 ± 0.06[b]	0.62 ± 0.08[a]
Average diameter (μm)	0.44 ± 0.08[d]	0.56 ± 0.16[b]	0.51 ± 0.15[c]	0.66 ± 0.09[a]
Eccentricity	0.56 ± 0.16[b]	0.70 ± 0.14[a]	0.57 ± 0.18[b]	0.43 ± 0.12[c]
Cross-sectional area (μm^2)	0.18 ± 0.06[d]	0.28 ± 0.15[b]	0.2 ± 0.13[c]	0.36 ± 0.09[a]
Volume (μm^3)	0.04 ± 0.02[c]	0.07 ± 0.05[a]	0.06 ± 0.04[b]	0.08 ± 0.03[a]
Mitochondrial cristae type	lamellar cristae	lamellar cristae	lamellar cristae	lamellar cristae

Note: the same parameter was compared during spermiogenesis, and the significant difference was labeled by different letters ($P < 0.05$).

Table S2 Primer sequences used for the cloning of *mfn2* cDNA, real-time quantitative PCR, and prokaryotic expression

Primers	Primer sequences (5' to 3')	Purpose
mfn2F	CAAAGGCTTGGGATGGCT	PCR for intermediate segment cloning
mfn2R	TCCAGATGGGAGATGCGTT	PCR for intermediate segment cloning
UPM-Long	CTAATACGACTCACTATAGGGCAAGCAGTGGTATCAACGCAGAGT	5' RACE
UPM-Short	CTAATACGACTCACTATAGGGC	5' RACE
NUP	AAGCAGTGGTATCAACGCAGAGT	5' RACE
5' MFN2R1	CATCAATGCCTGGGCTGTCTACTAATAC	5' RACE
5' MFN2R2	AGAGGGGATGTGGGTGAATGACGA	5' RACE
Outer Primer	TACCGTCGTTCCACTAGTGATTT	3' RACE
Inner Primer	CGCGGATCCTCCACTAGTGATTTCACTATAGG	3' RACE
3' MFN2F1	GCACTGGTTTATGTCTATGAGC	3' RACE
3' MFN2F2	CACAGAGAAGTACCTGACAGAGTC	3' RACE
qmfn2F	GGTCGTCATTCACCCACA	qPCR
qmfn2R	AGAGAGCAACTTTCATTCGG	qPCR
qgapdhF	CTGGTGAAGTTGGAGAAAAAG	qPCR
qgapdhR	GCTGAAGGAGCAGAGATGAT	qPCR
Anti-mfn2-F2	CGCGGATCCGCTGATGTGTTTGTCCTGGT	Prokaryotic expression
Anti-mfn2-R2	CCGCTCGAGGACTTTCTCCATTGTGCCC	Prokaryotic expression

Table S3 Primer sequences used for the cloning of *drp1* cDNA, real-time quantitative PCR, and prokaryotic expression

Primers	Primer sequences (5' to 3')	Purpose
drp1F	AGTTCGGTTTTGGAGAGCCT	PCR for intermediate segment cloning
drp1R	CACTGATTATTTGGCTGGCTT	PCR for intermediate segment cloning
UPM-Long	CTAATACGACTCACTATAGGGCAAGCAGTGGT ATCAACGCAGAGT	5' RACE
UPM-Short	CTAATACGACTCACTATAGGGC	5' RACE
NUP	AAGCAGTGGTATCAACGCAGAGT	5' RACE
5'drp1R1	GTCGGTGTTGGCAGCGGTGA	5' RACE
5'drp1R2	TTTCCCACTGCTCTGTGTACCAATTACT	5' RACE
Outer Primer	TACCGTCGTTCCACTAGTGATTT	3' RACE
Inner Primer	CGCGGATCCTCCACTAGTGATTTCACTATAGG	3' RACE
3'drp1F1	CCCCCACAGGAAGACAAGAATAGC	3’RACE
3'drp1F2	AGTCAACAGGCGTCAACCTTCTCG	3’RACE
qdrp1F	ATCTCCAACCCCAACTCTATC	qPCR
qdrp1R	CCAGTGTTCTCCTCCCATC	qPCR
qgapdhF	CTGGTGAAGTTGGAGAAAAAG	qPCR
qgapdhR	GCTGAAGGAGCAGAGATGAT	qPCR
Anti-Pedrp1-F1	CGCGGATCCAGTTCGGTTTTGGAGAGCC	Prokaryotic expression
Anti-Pedrp1-R1	CCGCTCGAGGTCCATCAGGTCAAGTTTAGT	Prokaryotic expression

Table S4 Tools used in this study to analyze protein structures and properties

Contents	Website or software
Primary structure	http://www.bio-soft.net/sms/
Molecular weight and isoelectric point	https://web.expasy.org/protparam/
Tertiary structure	https://zhanglab.ccmb.med.umich.edu/I-TASSER/
GTP binding sites	https://blast.ncbi.nlm.nih.gov/Bla st.cgi
Structural domain	https://www.ncbi.nlm.nih.gov/Structure/cdd/wrpsb.cgi, http://smart.embl-heidelberg.de and building by referencing structural domain of homologous proteins
Multiple sequence alignments	Vector NTI 11.5（Invitrogen，California，USA）
Evolutionary tree	MEGA 5.1（Informar Technologies，USA）

Table S5 GenBank accession numbers for MFN2 homologous proteins in multiple sequence alignments and neighbor-joining phylogenetic tree

Species	Accession number
Homo sapiens	AAH17061.1
Mus musculus	NP_001272849.1
Bos taurus	CBM40461.1
Parus major	XP_015503936.1
Taeniopygia guttata	XP_002194143.3
Catharus ustulatus	XP_032935534.1
Nanorana parkeri	XP_018425682.1
Python bivittatus	XP_007441486.1
Pelodiscus sinensis	XP_006127967.1
Xenopus tropicalis	NP_001016514.1
Danio rerio	NP_001121726.1
Acipenser ruthenus	XP_034760991.1
Clupea harengus	XP_012696382.1
Schistosoma japonicum	CAX69703.1
Echinococcus granulosus	EUB61133.1
Mizuhopecten yessoensis	OWF56197.1
Crassostrea gigas	XP_011449174.2
Nematostella vectensis	XP_032239660.1
Daphnia magna	KZS16364.1
Strongylocentrotus purpuratus	XP_030847518.1
Apostichopus japonicus	PIK61310.1
Hypsibius dujardini	GAV00492.1

Table S6 GenBank accession numbers for DRP1 homologous proteins in multiple sequence alignments and neighbor-joining phylogenetic tree

Species	Accession number
Homo sapiens	NP_036192.2
Mus musculus	NP_690029.2
Pygoscelis adeliae	XP_009331311.1
Gallus gallus	XP_015141156.1
Dermochelys coriacea	XP_043374384.1
Chelydra serpentina	KAG6939684.1
Xenopus laevis	XP_041440882.1
Bufo bufo	XP_040295010.1
Danio rerio	XP_005163105.1
Cyprinus carpio	XP_042608675.1
Drosophila melanogaster	NP_001259946.1
Vespa mandarinia	XP_035736130.1
Crassostrea gigas	XP_011428083.1
Mizuhopecten yessoensis	XP_021370324.1
Helobdella robusta	XP_009008795.1
Caenorhabditis elegans	AAD49861.1
Trichinella papuae	KRZ79511.1
Strongyloides ratti	CEF59341.1
